# Comprehensive analysis of epigenetics regulation, prognostic and the correlation with immune infiltrates of *GPX7* in adult gliomas

**DOI:** 10.1038/s41598-022-10114-1

**Published:** 2022-04-19

**Authors:** Wallax Augusto Silva Ferreira, Glauco Akelinghton Freire Vitiello, Tiago da Silva Medina, Edivaldo Herculano Correa de Oliveira

**Affiliations:** 1 Laboratory of Cytogenomics and Environmental Mutagenesis, Environment Section (SAMAM), Evandro Chagas Institute (IEC), Ananindeua, Brazil; 2 Translational Immuno-Oncology Group, International Research Center, A.C. Camargo Cancer Center, São Paulo, Brazil; 3 National Institute of Science and Technology in Oncogenomics and Therapeutic Innovation, São Paulo, Brazil; 4 Institute of Exact and Natural Sciences, Faculty of Natural Sciences, Federal University of Pará (UFPA), Belém, Brazil

**Keywords:** Epigenetics, Transcriptomics, Cancer, Bioinformatics, Gene expression analysis, Data mining, Databases, Gene ontology, Microarrays, DNA, RNA, Cancer metabolism, Cancer microenvironment, CNS cancer, Oncogenes, Tumour biomarkers, Tumour immunology, Biomarkers

## Abstract

Gliomas are the most commonly occurring malignant brain tumor characterized by an immunosuppressive microenvironment accompanied by profound epigenetic changes, thus influencing the prognosis. Glutathione peroxidase 7 (*GPX7*) is essential for regulating reactive oxygen species homeostasis under oxidative stress. However, little is known about the function of *GPX7* in gliomas. In this study, we hypothesized that *GPX7* methylation status could influence biological functions and local immune responses that ultimately impact prognosis in adult gliomas. We conducted an integrated bioinformatics analysis mining *GPX7* DNA methylation status, transcriptional and survival data of glioma patients. We discovered that *GPX7* was remarkably increased in glioma tissues and cell lines, and was associated with poor prognosis. This upregulation was significantly linked to clinicopathological and molecular features, besides being expressed in a cell cycle-dependent manner. Our results consistently demonstrated that upregulation of *GPX7* is tightly modulated by epigenetic processes, which also impacted the overall survival of patients with low-grade gliomas (LGG). Based on the analysis of biological functions, we found that *GPX7* might be involved in immune mechanisms involving both innate and adaptive immunity, type I interferon production and regulation of synaptic transmission in LGG, whereas in GBM, it is mainly related to metabolic regulation of mitochondrial dynamics. We also found that *GPX7* strongly correlates with immune cell infiltration and diverse immune cell markers, suggesting its role in tumor-specific immune response and in regulating the migration of immune cell types to the tumor microenvironment. Combining these multiple data, we provided the first evidence regarding the epigenetic-mediated regulatory mechanisms underlying *GPX7* activation in gliomas. Furthermore, our study brings key insights into the significant effect of *GPX7* in modulating both immune molecules and in immune cell infiltration in the microenvironment of gliomas, which might impact the patient outcome, opening up future opportunities to regulate the local immune response.

## Introduction

Gliomas are the most common primary intracranial tumor, representing up to 80% of all brain tumors^1^. They have aggressive behavior with a high mortality rate (median survival about 15 months), and exhibit notable variability at the histopathological and molecular level. According to the World Health Organization (WHO), they are categorized by the grade of malignancy (Grades 1, 2, 3 and 4), molecular markers (e.g., *IDH1/2* mutations, *TERT* mutations, 1p/19q co-deletion) and pathological features. As a result of an integrative view, gliomas are further segregated into 6 different families: (i) Adult-type diffuse gliomas (e.g., glioblastoma, IDH-wildtype); (ii) Pediatric-type diffuse low-grade gliomas (which have good prognosis); (iii) Pediatric-type diffuse high-grade gliomas (that behave aggressively); (iv) Circumscribed astrocytic gliomas; (v) Glioneuronal and neuronal tumors; and (vi) Ependymomas. Additionally, new molecular biomarkers have gained importance in providing both ancillary and defining diagnostic information, impacting clinical decision making and refining the WHO classification of an increasing number of gliomas^2,3^. As an example, the last update of WHO classification (5th edition) incorporated (i) *CDKN2A/B* homozygous deletion in *IDH*-mutant astrocytomas (covering grades 2–4), (ii) *TERT* promoter mutation, *EGFR* amplification, and + 7/− 10 copy number changes in *IDH*-wildtype diffuse astrocytomas as well as (iii) *MYB-* or *MYBL1* mutations in diffuse astrocytoma. However, despite the extensive molecular characterization, they remain incurable and reliable biomarkers are needed to further elucidate the molecular mechanism of glioma development^4^.

The endoplasmic reticulum-localized glutathione peroxidase 7 (*GPX7*), also known as non-selenocysteine phospholipid hydroperoxide glutathione peroxidase (NPHGPx), is a key PDI (protein disulfide isomerase) oxidase that uses H_2_O_2_ as a source of oxidative power^5^. *GPX7* acts as a critical intracellular sensor that detects redox level and transmits reactive oxygen species (ROS) signals to its interacting proteins (redox-sensitive thiol-containing proteins) by disulfide bonds shuttling, supporting multiple biologic processes such as oxidative protein folding^5–7^, the release of the non-targeting short interfering RNAs (siRNAs)-associated stress^8,9^ and protection of the organism against systemic oxidative stress^5,10–13^. Among the *GPX7*-interacting proteins, well-known examples include GRP78, CPEB2, XRN2, ADF, GRP75, HSP7C, ERp72, eEF1A-1, U-Tmod (TMOD3), ErJ3, and Histone H1b^8,14^. Some studies have generated compelling evidence for the relevance of *GPX7* in metabolic diseases^12,15,16^, neurodegeneration^17–19^, viral infection^20^ and cardiovascular diseases^8^. In addition, dysregulation of *GPX7* has been found to contribute in different ways to the tumorigenesis and progression of many human carcinomas, such as esophageal adenocarcinoma^21^, gastric cancer^22^, hepatocellular carcinoma^23^, acute myeloid leukemia^24^ and breast cancer^25^. On the other hand, the role of *GPX7* in the development of gliomas and the epigenetic mechanisms underlying this process have yet to be fully characterized. To address this issue, in the present study, we used database research and bioinformatic analyses to assess the expression of *GPX7* in gliomas and analyze its epigenetic modulation, potential biological functions, prognostic value and correlation with tumor-infiltrating immune cells.

## Material and methods

### Oncomine database analysis

Using the Oncomine database (https://www.oncomine.org)^26^, we examined the expression differences of *GPX7* expression across several human tumors and the corresponding normal tissues. The threshold for all analyses was defined as follows: Fold change = 2.5; Gene ranking: top 10%; Data type: mRNA, and “Analysis type”: cancer vs. normal analysis. All statistical methods and values were obtained directly from the corresponding database. The student’s t-test was used to generate the p-value for expression differences and *p* < 0.05 was considered statistically significant. All microarray platforms were considered.

### Differential gene expression analysis

To validate our findings of the differential expression found for glioma tumors in the Oncomine database, we analyzed *GPX7* expression using The Cancer Genome Atlas (TCGA) RNA-seq data (LGG = 518 samples; GBM = 163 samples; Genotype-Tissue Expression (GTEx) dataset as a control dataset, N = 207 samples), Chinese Glioma Genome Atlas (CGGA) RNA-seq data (Normal samples = 20 samples; LGG = 426 samples; GBM = 225 samples), Rembrandt microarray dataset^27^ (Normal samples = 28 samples; LGG samples = 225 samples; GBM samples = 219 samples), Gravendeel microarray dataset^28^ (Normal samples = 8 samples; LGG = 117 samples; GBM = 159 samples), and Kamoun microarray cohort^29^ (Normal samples = 9 samples; LGG = 154 samples; GBM = 16 samples). Clinical and molecular information was downloaded from CGGA (http://www.cgga.org.cn)^30^, GlioVis (http://gliovis.bioinfo.cnio.es/)^31^ and GDC (Genomic Data Commons).

### Cancer cell line encyclopedia database analysis

We expanded our analysis by evaluating the *GPX7* expression in a panel with multiple human cancer cell lines (including 66 glioma cell lines) using data from the Cancer Cell Line Encyclopedia (CCLE, https://portals.broadinstitute.org/ccle/)^32,33^, which spans multidimensional arrays and RNA-seq datasets for over 1457 human cancer cell lines. The expression was ranked using Affymetrix GeneChip data.

### Analysis of gene expression omnibus (GEO) microarray datasets

The Gene Expression Omnibus (GEO) (https://www.ncbi.nlm.nih.gov/gds)^34^ is a public database of the National Center of Biotechnology Information that stores high-throughput gene expression datasets. To profile the variation of *GPX7* expression during the cell cycle in T98G cells (Human GBM Cell line), we downloaded and analyzed the GSE8537 dataset^35^, which was based on the Affymetrix GPL570 platform ([HG-U133-Plus2] Human Genome U133 Plus 2.0 Array).

Also, we obtained *GPX7* gene expression profiling from three therapeutically relevant transcriptome microarrays: (i) GSE43452 dataset^36^, from a human GBM cell line (U87MG) treated with temozolomide at 20 µM for 24 h, based on the Illumina GPL10558 (HumanHT-12 V4.0 expression beadchip); (ii) GSE39223 dataset^37^, that included U87MG-EV human glioblastoma xenograft tumor treated with bevacizumab (10 mg/kg of body weight), which was subcutaneously implanted into 7 to 8-week-old female BALB/c SCID mice (Harlan Sprague Dawley, Inc., Indiana), 100μL of cell suspension mixed with an equal volume of matrigel (BD Bioscience). After 3 days, the second dose of Bevacizumab was given and, 4 h later, mice were sacrificed and the tumors were collected. This dataset was based on the Affymetrix GPL70 platform ([HG-U133-Plus2] Human Genome U133 Plus 2.0 Array); and (iii) GDS2428 dataset^38^, from short-term cultured glioblastoma cells (GLI56) treated with 5-aza-2′-deoxycytidine (5-aza-dC) 5 μM for 96 h to induce DNA demethylation.

### UCSC (University of California Santa Cruz) Xena Browser

Heat map of the integrated analysis with clinical information (Histology and WHO grade), copy number alterations (CNAs) and expression levels of *GPX7* were obtained by mining TCGA-LGG-GBM dataset (1153 samples) by using the UCSC Xena Browser (https://xena.ucsc.edu/). The gene expression profile from RNAseq data was indicated as normalized_log2[norm_count + 1] and CNAs status as Log2 (Tumor/Normal).

### Clinical correlations and survival analysis

To perform the correlation between *GPX7* expression and clinicopathological/molecular parameters, gene expression of normalized and pre-processed data (both microarray and RNA-seq) of the TCGA-LGG-GBM dataset and CGGA-LGG-GBM was downloaded from GlioVis (http://gliovis.bioinfo.cnio.es/)^31^. The latest corresponding clinical data were downloaded from GDC (Genomic Data Commons), and patients with unavailable clinical information were excluded. All statistical analyses were performed by R v4.0.5 (https://www.r-project.org/).

The disease-free survival (DFS) and overall survival (OS) curves for TCGA-LGG and TCGA-GBM cohorts and clinicopathological subgroups were conducted using the GEPIA tool (http://gepia.cancer-pku.cn)^39,40^. Patients were dichotomized into two groups (Red: high expression; Blue: low expression) according to the median expression level of *GPX7*. All survival curves were generated by the Kaplan–Meier methods. The log-rank *P*-value and hazard ratio (HR) with 95% confidence intervals were also calculated. The survival findings were further validated in two different glioma cohorts: (i) Chinese Glioma Genome Atlas (CGGA) (http://www.cgga.org.cn/) (n = 325 samples)^41^; and (ii) Repository of Molecular Brain Neoplasia Data (Rembrandt) (n = 444 samples)^42–44^.

Finally, ROC Plotter (http://www.rocplot.org/)^45^ was used to assess the sensitivity and specificity of *GPX7* for classifying chemotherapy responsiveness in GBM patients (n = 454). We checked all included GBM patients receiving “any chemotherapy” as the primary analysis with the secondary analysis for: Bevacizumab, Irinotecan, Nitrosoureas (Lomustine, Carmustine, Estramustine, Laromustine, Nimustine), Topoisomerase inhibitor, Angiogenesis inhibitor, Carmustine, Lomustine and Temozolomide. A box plot displaying *GPX7* expression levels in responders and non-responders (based on OS at 16 months) was provided along with the area under the curve (AUC) and their respective *p*-values. The differences were assessed with the Mann–Whitney *U *test. No additional filter was applied, and no outliers were excluded from the analysis.

### GO enrichment analysis of *GPX7*-related genes in LGG and GBM based on the LinkedOmics database

To evaluate the potential functional mechanism of *GPX7* in gliomas, we obtained all genes co-expressed with *GPX7* from the LinkedOmics (http://www.linkedomics.org/)^46,47^, using the LinkFinder module. Spearman’s test was conducted to perform statistical analyses. To derive biological insights from the association results, the gene ontology (GO) enrichment analysis was conducted by Gene Set Enrichment Analysis (GSEA) with a minimum number of genes (size) of 10 and the simulation of 1000. A *p*-value  < 0.05 was deemed to indicate statistical significance.

### *GPX7 *Methylation analysis

The methylation levels (β-value) of CpG sites associated with *GPX7* in TCGA-LGG and TCGA-GBM were assessed using the bioinformatics platform MethSurv (https://biit.cs.ut.ee/methsurv/)^48^. Moreover, the overall survival (OS) analysis for each CpG site was assessed using Kaplan–Meier plots. Log‐rank tests were used to measure the statistical significance and Log‐rank *p* < 0.05 was considered significant. The methylation pattern of each probe indicating subregions of the query gene was plotted using a heatmap by ClustVis (https://biit.cs.ut.ee/clustvis/)^49^.

Thereafter, Shiny Methylation Analysis Resource Tool (SMART; http://www.bioinfo-zs.com/smartapp/)^50^ was used to determine the correlation between the degree methylation of each probe (β-value) and the corresponding expression level of *GPX7* in both TCGA-LGG and TCGA-GBM cohorts. The threshold of all analyses was determined as follows: Aggregation method: mean; Correlation coefficient: spearman.

### *GPX7*-associated microRNAs

To predict *GPX7*-targeting mirRNAs we used (i) cancer regulome (http://explorer.cancerregulome.org/) (correlation of Abs = 0.4; Max results: 200; p < 0.05 ); (ii) DIANA-miRPath v.3 (http://snf-515788.vm.okeanos.grnet.gr/)^51^; (iii) Targetscan (http://www.targetscan.org/)^52^; (iv) miRWalk 3.0 database (http://mirwalk.umm.uni-heidelberg.de/)^53,54^; and (iv) miRDB database (http://mirdb.org/)^55–57^. All candidate miRNAs were compared with all databases. To understand the signaling pathways associated the miRNA signatures, we performed the Kyoto Encyclopedia of Genes and Genomes (KEGG) pathway enrichment analysis^58–60^ in DIANA-mirPath v.3. *p* < 0.05 and false discovery rate (FDR) were considered to estimate statistical significance in enrichment analysis.

### Tumor immune estimation resource (TIMER) analysis

The TIMER database (http://timer.comp-genomics.org/)^61,62^ was used to estimate the correlations between *GPX7* expression and the abundance of immune cell infiltration levels. It uses a deconvolution method to deduce the abundance of tumor-infiltrating immune cells based on gene expression profiles. In this study, we adopted CIBERSORT, CIBERSORT-ABS, EPIC, quanTIseq, TIMER, xCell and MCPcounter immune deconvolution algorithms to cover as many immune cell types as possible. Tumor purity was considered when calculating Spearman’s correlation, and a *p*-value <0.05  was considered statistically significant.

### TISIDB database analysis

TISIDB is an integrated repository web portal to analyze interactions between tumors and the immune system^63^. It integrates multiple types of data resources in oncoimmunology, including literature mining results from the PubMed database and TCGA. The TISIDB was used to assess the role of *GPX7* in the tumor–immune interplay.

### Statistical analysis

R software (v4.0.5) was employed to implement the statistical analyses of the study. *p* values < 0.05 were considered significant unless otherwise specified. The relationships of *GPX7* expression and clinicopathological features were estimated using the Chi-Squared Test. Analyses with more than two groups were performed by one-way ANOVA test. Statistical significance was denoted by *p < 0.05, **p < 0.01, ***p < 0.001. The associations of the gene expression levels were analyzed using a non-parametric Spearman’s rho test. The strength of the correlation was determined using the following: (r) ≥ 0.7 indicated a strong correlation; r < 0.7 and r ≥ 0.3 indicated a moderate correlation, and r < 0.3 indicated a weak correlation.

## Results

### *GPX7* is overexpressed in gliomas tissues and cell lines

**Figure 1:**
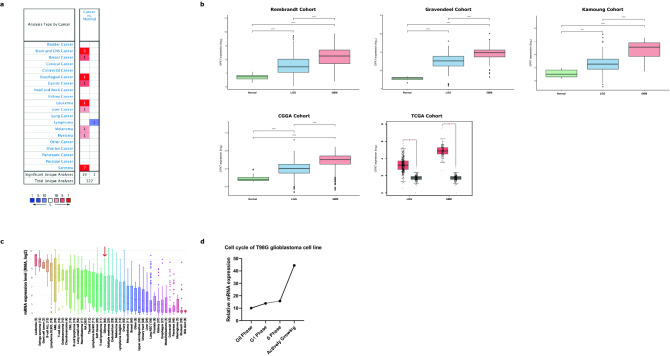
Transcription levels of *GPX7* in different types of human cancers compared with normal tissues (Oncomine database). (**a**) The number of datasets with statistically significant mRNA upregulated (red) or downregulated expression (blue) of *GPX7*. The numbers in the colored cell represent datasets meeting the threshold (see “Material and methods”). The gene rank was analyzed by the percentile of the target gene at the top of all genes measured in each research. The cell color is determined by the best gene rank percentile for the analyses within the cell. Significant Unique Analyses indicate that the queried gene is significantly different in studies. Total Unique Analysis indicates the total number of studies that contain the queried gene. (**b**) *GPX7* expression levels in distinct glioma cohorts. Rembrandt microarray cohort (Normal samples = 28 samples; LGG samples = 225 samples; GBM samples = 219 samples); Gravendeel microarray cohort (Normal samples = 8 samples; LGG = 117 samples; GBM = 159 samples); Kamoun microarray cohort (Normal samples = 9 samples; LGG = 154 samples; GBM = 16 samples); Chinese Glioma Genome Atlas (CGGA) RNA-seq cohort (Normal samples = 20 samples; LGG = 625 samples; GBM = 388 samples); TCGA RNA-seq cohort (LGG = 518 samples; GBM = 163 samples). Red boxplots: Tumor samples from TCGA. Grey boxplots: Normal samples from GTEx (Genotype–Tissue Expression database) (N = 207 samples). (**c**) mRNA expression of *GPX7* in different types of cell lines of human cancer from CCLE (Cancer cell line encyclopedia). The red arrow indicates the glioma cell lines (66 cell lines). The number next to the lineage name represents the number of cell lines in the lineage. The expression ranks in gliomas using Affymetrix GeneChip data. Statistical significance is denoted by *p < 0.05, **p < 0.01, ***p < 0.001. (**d**) *GPX7* gene expression profiling during the cell cycle in T98G cells (Human Glioblastoma Cell line) (GDS3364 from GSE8537 dataset)^35^.

Changes in expression levels of most glutathione peroxidases have been reported in several tumors. However, to the best of our knowledge, few studies have investigated the *GPX7* expression and its impact on most cancers. To this end, we explored the expression patterns of *GPX7* from a pan-cancer perspective, using microarray data through the differential analysis tool of the Oncomine database. Strikingly, we observed that *GPX7* was overexpressed in 19 datasets (brain, breast, esophageal, gastric, liver cancers, leukemia, melanoma, myeloma and sarcoma) and underexpressed in lymphomas (Fig. 1a), indicating that the dysregulation of this glutathione peroxidase was a common phenomenon across several tumors.

Subsequently, we focused our analysis on the differential expression of *GPX7* in all brain cancer datasets deposited on Oncomine. As presented in Table 1, *GPX7* was found to be higher in gliomas, especially in astrocytic, oligodendroglial and mixed gliomas. To further expand and validate these results, we analyzed five transcriptome datasets: Rembrandt microarray^27^, Gravendeel microarray^28^, Kamoun microarray^29^, CGGA RNA-seq^30^, and TCGA RNA-seq. As shown in Fig. 1b, we consistently showed that *GPX7* was overexpressed in gliomas compared to normal samples in all datasets.

**Table 1:** Significant changes of *GPX7* expression among different types of glioma tumors and normal brain tissues (Oncomine database).

Gene	Gliomas tumors	Subtypes	WHO Grade	Fold change	*t*-test	*p* value	References
*GPX7*	Astrocytic	Pilocytic Astrocytoma	1	3.876	2.772	0.045	^64^
		Diffuse Astrocytoma	2	1.74	3.173	0.009	^65^
		Anaplastic Astrocytoma	3	1.785	7.031	9.79E-08	^65^
		Glioblastoma	4	3.046	12.484	4.52E-06	^66^
		Glioblastoma	4	1.335	4.75	4.01E-05	^67^
		Glioblastoma	4	2.391	14.416	6.66E-26	^65^
	Oligodendroglial	Oligodendroglioma	–	1.497	5.642	1.70E-07	^65^
		Anaplastic Oligodendroglioma	3	2.021	5.523	3.90E-06	^68^
	Mixed	Anaplastic Oligoastrocytoma	3	3.267	4.205	0.011	^68^

By assembling the Cancer Cell Line Encyclopedia (CCLE), we further explored whether the overexpression pattern of *GPX7* detected in LGG and GBM tissues would be similar in glioma cell lines. Accordingly, the vast majority of glioma cell lines highly expressed *GPX7* (Fig. 1c) and this overexpression was cell cycle-dependent manner (Fig. 1d).

Together, these data support that *GPX7* overexpression may be involved in glioma tumorigenesis, which appears to be differentially regulated by molecular mechanisms during the progression of the cell cycle, perhaps being modulated by various factors of the tumor microenvironment, especially in response to exposure to oxidative stress (ROS accumulation). These data prompted us to further investigate the relationship between the expression of this gene with the clinical and molecular parameters in LGG and GBM.

### Clinicopathological and molecular features of adult LGG and GBM strongly impacts *GPX7* expression

**Figure 2:**
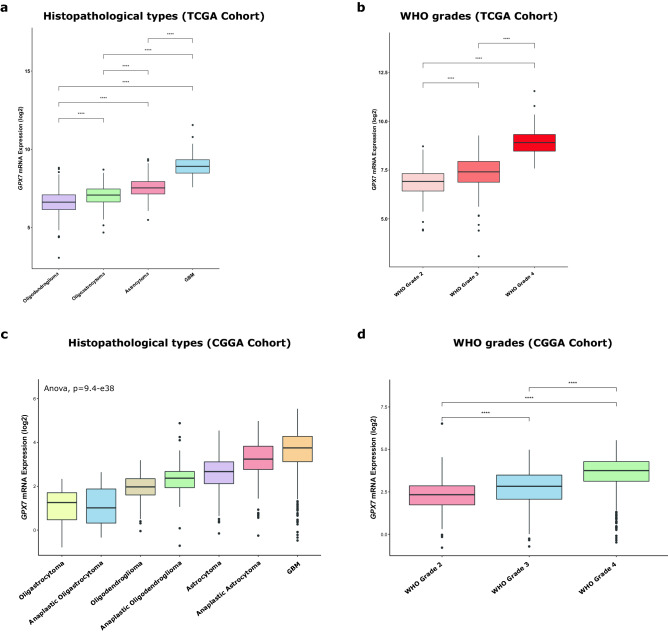
*GPX7* is highly expressed in gliomas and is significantly associated with the advanced stage of tumors. (**a**) Relative mRNA expression of *GPX7* in histopathological types of gliomas; (**b**) Relative mRNA expression of *GPX7* in different gliomas WHO grades (2, 3 and 4) from the TCGA cohort; (**c**) Relative mRNA expression of *GPX7* in histopathological types of gliomas from the CGGA cohort; (**d**) Relative mRNA expression of *GPX7* in different gliomas WHO grades (2, 3 and 4) from the CGGA cohort. Statistical significance was denoted by asterisks: *p ≤ 0.05; **p ≤ 0.01; ***p ≤ 0.001; ****p ≤ 0.0001.

Leveraging the TCGA and CGGA datasets, we next explored the correlation between *GPX7* expression and the clinicopathological/molecular parameters of gliomas (WHO grade 2–4). Notably, in both cohorts, *GPX7* was prominently higher in GBM than in LGG tumors (Oligoastrocytoma; Oligodendroglioma; Astrocytoma), while the expression of *GPX7* was significantly higher as pathological grade increased (Fig. 2a–d). Besides, *GPX7* expression was found to be significantly correlated with additional pharmaceutical therapy (*p* = 0.0054), primary therapy outcome success (*p* = 0.000038), new tumor event after initial treatment (*p* = 0.0000029), Postoperative rx tx (*p* = 0.038), radiation therapy (*p* = 0.0000000013), sensory changes (*p* = 0.0027) and visual changes (*p* = 0.01) (Supplementary Table S1) in TCGA cohort. Furthermore, in the CGGA dataset, the elevated expression of *GPX7* was correlated with radiochemotherapy status and progression status, consistent with the findings seen in the TCGA dataset (Supplementary Table S2). In view of the aforementioned findings, *GPX7* is abundantly expressed in aggressive gliomas, suggesting that *GPX7* might be involved in the malignant progression of gliomas.

**Figure 3:**
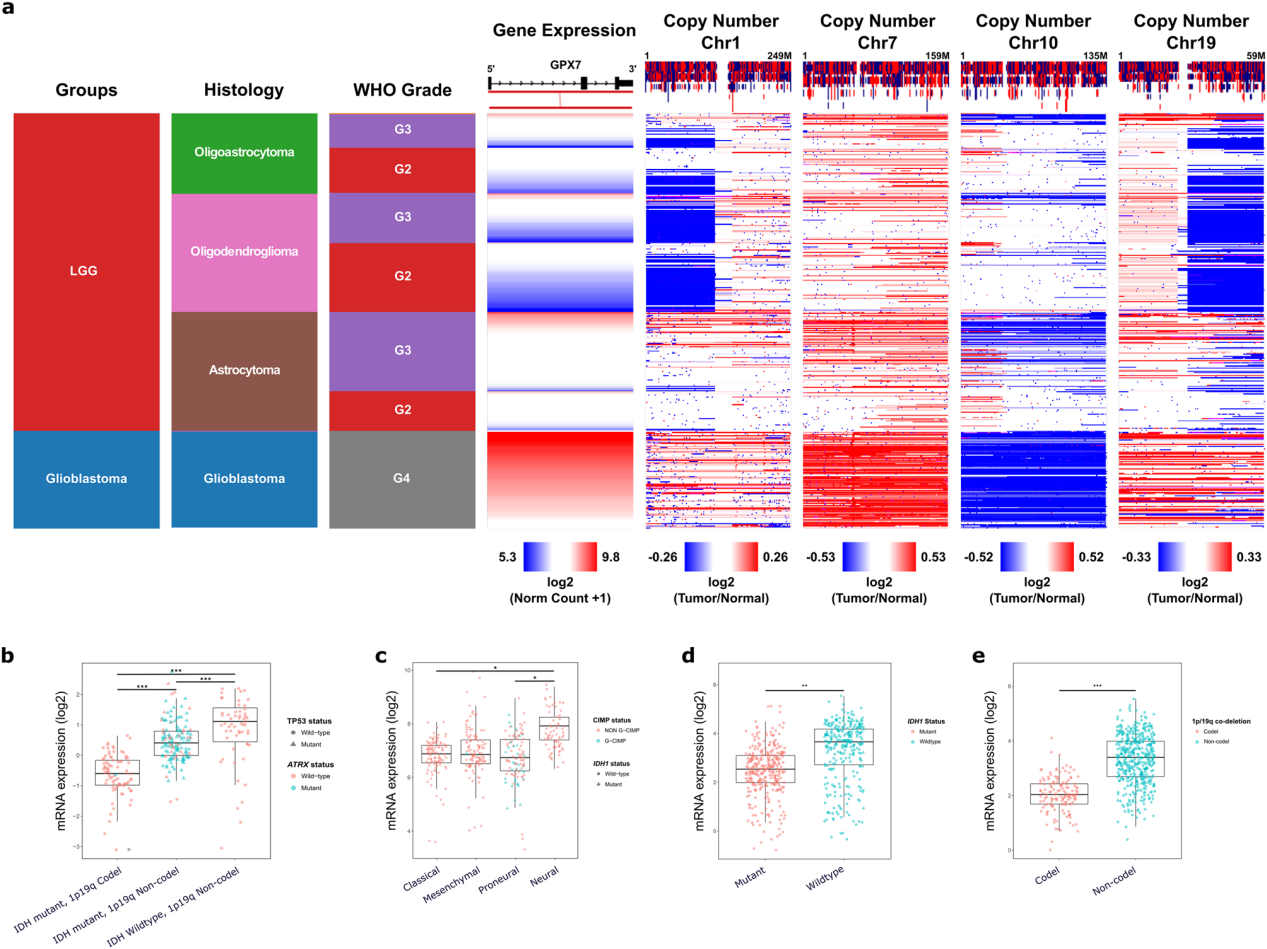
*GPX7* expression was correlated with CNAs (Copy Number Alterations), *IDH1* mutational status and 1p/19q co-deletion. (**a**) Heatmap integrating *GPX7* expression (Log2—Norm Count + 1), CNAs (Copy Number Alterations) status (Log2—Tumor/Normal) of chromosomes 1, 7, 10 and 19, histology and WHO grade of LGG and GBM cohorts from TCGA. *GPX7* mRNA expression in molecular subtypes of (**b**) Low-Grade Gliomas (LGG) cohort, according to WHO^81^ (*IDH*-mutant, 1p/19q codeleted; *IDH*-mutant, 1p/19q non-codeleted; and *IDH*-wildtype, 1p/19q non-codeleted group) and of (**c**) GBM cohort, according to Verhaak^82^ (Proneural; Classical; Mesenchymal; Neural) from TCGA database. (**d**) *GPX7* expression correlates with *IDH-1* status in CGGA gliomas. (**e**) *GPX7* expression correlates with 1p/19q co-deletion in CGGA gliomas. *p < 0.05, **p < 0.01, ***p < 0.001.

Gliomas, like other tumors, show high genetic heterogeneity^69–72^. Numerous studies revealed that the most common somatic chromosomal changes in these tumors are complete or partial loss of chromosomes 1, 7, 10 and 19^73–80^. Intriguingly, our data showed that higher *GPX7* expression was significantly correlated with 1p/19q non-codeleted group, carrying concomitant gain of chr7/loss of chr10, mostly seen in GBM patients (Fig. 3a; Supplementary Fig. S1). These findings led us to hypothesize that the molecular classification proposed for LGG and GBM could also impact *GPX7* expression.

The most recent guidelines for the classification of gliomas recommend that LGG should be categorized into three major groups^81^: (1) *IDH*-mutated 1p/19q codeleted group; (2) *IDH*-mutated 1p/19q non-codeleted group (most of them have mutations in *TP53* and *ATRX*); (3) *IDH*-wildtype 1p/19q non-codeleted group. Here, using this classification, we showed that *GPX7* was significantly higher in the TCGA-LGG patients with wild-type *IDH1*, *TP53* and *ATRX*, without 1p/19q non-codeletion (Fig. 3b). Subsequently, using the TCGA-GBM cohort, we considered the molecular subtypes proposed by Verhaak (Proneural; Classical; Mesenchymal; Neural)^82^ as well as the *IDH1* status, *MGMT* methylation status, and G-CIMP (Glioma CpG island methylator phenotype) status. As noted in Supplementary Fig. S2, *GPX7* expression varied considerably depending on the CIMP status and *IDH1* status. Remarkably, *GPX7* was increased in wildtype *IDH1* non-GCIMP neural GBMs (Fig. 3c). To validate our results, CGGA samples were subsequently used to verify these relationships. *GPX7* expression was also significantly associated with *IDH* mutation status (Fig. 3d) and 1p/19q co-deletion status (Fig. 3e).

Lastly, concerning the most commonly mutated genes in LGG, wild-type *TERT*, *IDH1/2*, *CIC*, *NOTCH1, PIK3CA* and *FUBP1* were significantly enriched in TCGA-LGG patients with high expression of *GPX7* (Supplementary Fig. S3a–g). Additionally, patients harboring *TP53, ATRX, NF1, PTEN* and *EGFR* mutations also over-expressed *GPX7* (Supplementary Fig. S3h–l).

In summary, our results indicate that the overall *GPX7* expression varied significantly as a function of histopathological grade and is highly associated with glioma progression. Also, *GPX7* expression might be correlated to chromosomal changes, mainly concomitant gain of chr7/loss of chr10 (primarily seen in GBM), as well as mutations in *TP53, ATRX, NF1, PTEN* and *EGFR* (mostly observed in LGG).

### The overexpression of *GPX7* correlates with unfavorable prognosis in adult LGG tumors

**Figure 4:**
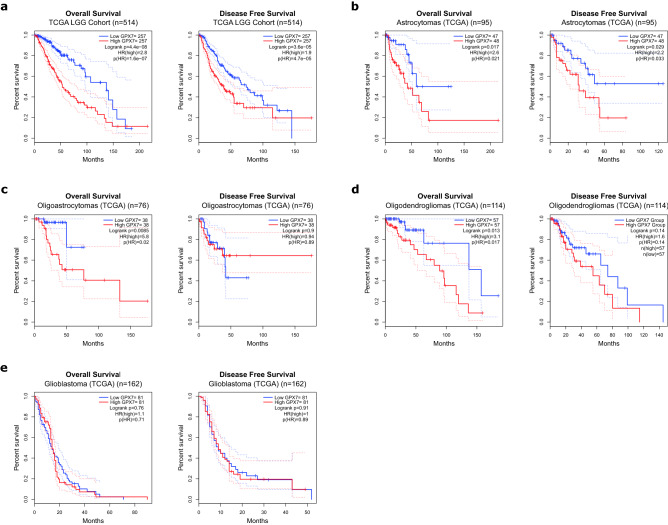
Kaplan–Meier survival curves are based on high (red) and low (blue) expression of *GPX7* in LGG and GBM from the TCGA database*.* Overall survival (OS) (left) and disease-free survival (DFS) (right) of (**a**) All histological subgroups of LGG (n = 514), (**b**) Astrocytomas (n = 95), (**c**) Oligoastrocytomas (n = 76), (**d**) Oligodendrogliomas (n = 114) and (**e**) Glioblastomas (n = 162). X-axis: patients’ survival duration (months); Y-axis: patients’ survival rate. *p*-Values for all survival analyses have been calculated using the log-rank test.

Next, we applied Kaplan–Meier survival curves to assess the *GPX7* prognostic role in TCGA LGG and GBM cohorts. The results illustrated that higher expression of *GPX7* was markedly correlated with worse overall survival (OS) (HR = 2.8; Logrank *p* = 4.4e–08) and disease-free survival (DFS) (HR = 1.9, Logrank *p* = 3.6e–05) in LGG (Fig. 4a). Furthermore, considering all histological subgroups of LGG, higher *GPX7* was significantly correlated with poor OS in astrocytomas (HR = 2.6; Logrank *p* = 0.017) (Fig. 4b), oligoastrocytomas (HR = 5.8; Logrank *p* = 0.0085) (Fig. 4c) and oligodendrogliomas (HR = 3.1; Logrank *p* = 0.013) (Fig. 4d) and poor DFS in astrocytomas (HR = 2.2; Logrank *p* = 0.029) (Fig. 4b). However, no significant correlation was observed between the expression of *GPX7* and the prognosis of GBM (Fig. 4e). Thus, we naturally extended our analysis to other glioma datasets, and in all of them, we consistently found that overexpression of *GPX7* was associated with a worse prognosis (Supplementary Fig. S4–S5). In conclusion, these data suggested that *GPX7* had a significant effect on the prognosis of LGG.

### Hypomethylation of *GPX7* promotes its expression in LGG tumors

**Figure 5:**
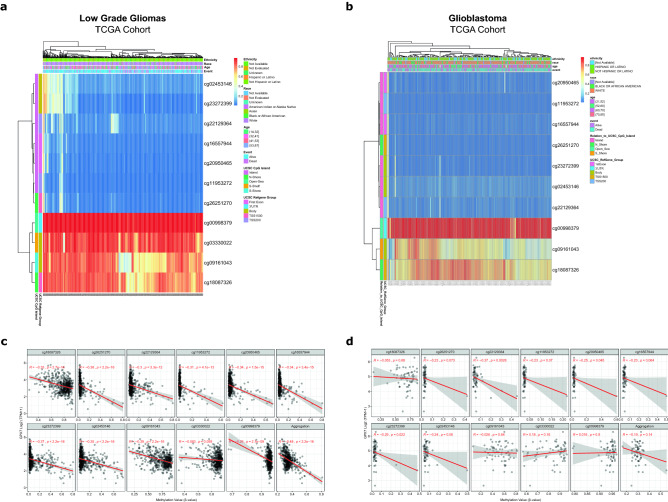
Dynamics of the DNA methylation across CpG sites of *GPX7* in LGG and GBM tumors*.* (**a**) Heat-map showing the methylation levels of *GPX7* among different CpGs sites (probes) integrating ethnicity, race, age, vital status and genomic regions of CpG sites from LGG; (**b**) Heat-map showing the methylation levels of *GPX7* among different CpGs sites integrating ethnicity, race, age, vital status and genomic regions of CpG sites from GBM. (**c**) Pearson correlation between the degree of methylation (β-value) of each CpG site of *GPX7* and its expression level (Log2 – TPM + 1) in LGG; Aggregation method: mean; (**d**) Pearson correlation between the degree of methylation (β-value) of each CpG site of *GPX7* and its expression level (Log2 – TPM + 1) in GBM. Aggregation method: mean.

DNA methylation is one of the best-characterized epigenetic modifications generally occurring on cytosines that precede a guanine, which are concentrated in large clusters throughout the genome, called CpG islands. Consequently, DNA methylation can impact DNA conformation, chromatin structure, DNA–protein interactions, DNA stability and can modify gene expression. Furthermore, both initiation and late stages of oncogenesis may be led by DNA methylation changes, given that epigenetic changes have been demonstrated in multiple cancers, including gliomas^83,84^. In this regard, we next sought to uncover the molecular mechanisms that contribute to the high expression of *GPX7* in gliomas. For this, we analyzed the methylation levels of all CpG islands based on the regions defined by the UCSC Genome Browser (First Exon, 3’UTR, Body, TSS1500 and TSS200), S-Shore (2000 bp region 3′ adjacent to CpG island), S-Shelf (2000 bp region 3′ adjacent to S-Shore), and N-Shore (2000 bp region 5′ adjacent to CpG island), of LGG and GBM cohorts from TCGA database. Overall, both cohorts exhibited extensive hypomethylation levels at cg02453146, cg23272399, cg22129364, cg16557944, cg20950465, cg11953272 and cg26251270 CpG sites and higher hypermethylation levels at cg00998379 CpG site (Fig. 5a, b; Supplementary Table S3). Curiously, four regions (cg02453146, cg23272399, cg09161043 and cg18087326) from LGG stood out forming clusters with distinct methylation patterns, which might reflect the complexity/heterogeneity of the methylation program involved in these tumors.

Considering the aforementioned results, we sought to ascertain whether hypomethylation was the cause of increased *GPX7* expression. We assessed the levels of *GPX7* mRNA and correlated them with the methylation status of each CpG site (Fig. 5c, d). Strikingly, the methylation levels for all CpG sites revealed a significant negative correlation with mRNA levels in LGG tumors (Aggregation *ρ* = − 0.48, *p* < 2.2e−16) (Fig. 5c). To further strengthen these results, analyzing an independent dataset from GEO database, we verified that short-term exposure (96 h) of cultured glioblastoma cell line (GLI56) to 5-aza-2′-deoxycytidine (5-aza-dC) [5 μM], a global DNA demethylating agent, induced a substantial increase in the *GPX7* expression (Supplementary Fig. S6), thus strongly suggesting that upregulation of *GPX7* is tightly regulated by DNA methylation.

Since we confirmed the correlation between methylation and regulation of *GPX7* mRNA expression, we next analyzed the impact of the methylation pattern of each CpG site on overall survival (OS) in LGG patients. Consistently, seven CpG sites (cg02453146; cg23272399; cg11953272; cg03330022; cg09161043) distributed in different genomic regions (Body; TSS200; TSS1500) were significantly correlated with the prognosis of LGG (Supplementary Table S3). This evidence suggests that upregulation of *GPX7* was caused by DNA hypomethylation, with an impact on poor OS in patients with LGG.

### *GPX7* overexpression is highly correlated with histone acetyltransferase 1 (HAT1) expression and with H3K9ac and H3K27ac marks

It is well known that the crosstalk between both DNA hypomethylation and histone acetylation is involved in the upregulation of gene transcription^85–87^. In particular, histone acetylation, which is catalyzed by histone acetyltransferases (HATs), disrupts the electrostatic interaction between histones and DNA, thus conferring positive effects on gene expression. Based on cell localization, HATs are subdivided into (i) Type A HATs (mainly expressed in the nucleus) and (ii) Type B HATs (mainly expressed in the cytoplasm)^87,88^. Therefore, we speculate that *GPX7* expression could be correlated with HATs and acetylation marks in LGG and GBM.

**Figure 6:**
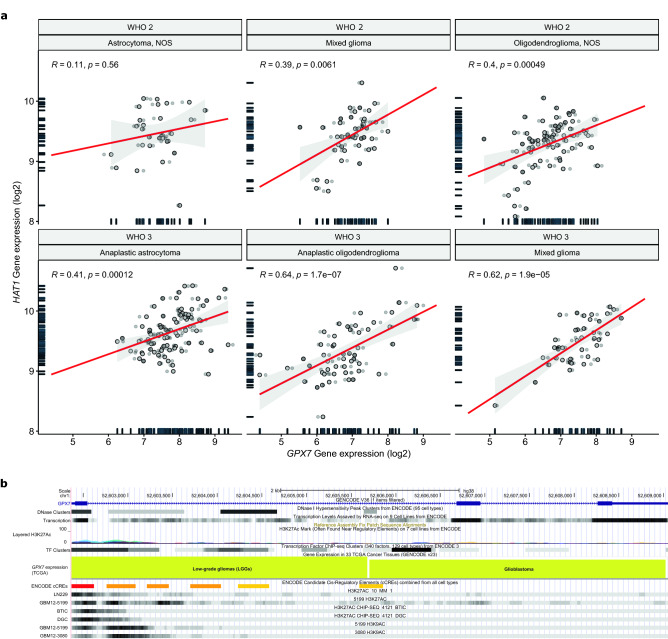
H3K9ac and H3K27ac marks can alter *GPX7* expression. (**a**) Correlation between *GPX7* and *HAT1* expression according to WHO grades (2 and 3) and histology of TCGA-LGG tumors. NOS: Not Otherwise Specified. (**b**) Representative UCSC genome browser view of *GPX7* genomic region showing chromatin immunoprecipitation (ChIP) enrichment for H3K9ac and H3K27ac (active chromatin marks) using ChIP-seq data from LN229 (GBM cell line) (GSE109340), GBM12-5199 (PDX model of GBM)^89^, GBM12-3080 (PDX model of GBM)^89^, GBM12-5199 (PDX model of GBM)^89^, GBM12-3080 (PDX model of GBM)^89^, Brain tumor initiating cells (BTICs)^90^, differentiated glioma cells (DGCs)^90^. **DNase clusters:** shows chromatin accessibility following binding of trans-acting factors in place of a canonical nucleosome. The display for this track shows site location and signal value as grayscale-colored items where higher signal values correspond to darker-colored blocks. This track is a composite annotation track containing multiple subtracks, one for each cell type. **Layered H3K27Ac:** This track shows the enrichment levels of the H3K27Ac histone mark across the genome as determined by a ChIP-seq assay. Also, this mark is thought to enhance transcription, possibly by blocking the spread of the repressive histone mark H3K27Me3. ***GPX7* expression (TCGA):** shows RNA expression level for each TCGA tumor (LGG and GBM) in GENCODE canonical genes. The gene scores are a total of all transcripts in that gene. **TF Clusters:** This track shows transcription factor (TF) binding sites derived from a large collection of ChIP-seq experiments. A gray box encloses each peak cluster of transcription factor occupancy. The darkness of the box is proportional to the maximum signal strength observed in any cell type contributing to the cluster. **ENCODE cCRES track:** This track displays the ENCODE Registry of candidate cis-Regulatory Elements (cCREs) in the mouse genome. cCREs are the subset of representative DNase hypersensitive sites across ENCODE samples supported by either histone modifications (H3K4me3 and H3K27ac) or CTCF-binding data. CCREs are colored and labeled according to classification by regulatory signature: Red (promoter-like signature); orange (proximal enhancer-like signature); yellow (distal enhancer-like signature); pink (DNase-H3K4me3); blue (CTCF-only).

Initially, we observed that most HATs were differentially expressed in LGGs and correlations between HATs and *GPX7* expression were mostly seen in LGG (Supplementary Fig. S7 and Supplementary Table S4). Notably, in both cohorts, the highest positive correlation was with histone acetyltransferase 1 (*HAT1*) (**LGG:** *ρ* = 0.71, *p* = 1.00e–51; **GBM:** *ρ* = 0.31; *p* < 5.50e–05) and among the LGG, this correlation was significantly higher in anaplastic oligodendrogliomas (WHO grade 3) (*ρ* = 0.64, *p* = 1.7e−07) and in mixed glioma (WHO grade 3) (*ρ* = 0.62, *p* = 1.9e−05) (Fig. 6a), suggesting that *GPX7* might be epigenetically regulated by HAT1-mediated acetylation, especially in high-grades.

Next, to directly show a functional link between the expression of *GPX7* and the acetylation of histones, we analyzed the distributions of active chromatin marks, marked by histone 3 lysine 9 and/or lysine 27 acetylation (H3K9ac or H3K27ac, respectively), mapped by ChIP-Seq using data from the ENCODE Project in UCSC Genome Browser database in different human glioma models. Strikingly, H3K9ac and H3K27ac marks were predominantly enriched near of active regulatory elements and in the TSS region of the *GPX7* in all models*,* as indicated by the colocalization of DNase clusters (DNaseI hypersensitivity), transcription factors (TF clusters) peaks and ENCODE cCREs peaks, which also correlates with an increase in *GPX7* expression (Fig. 6b), suggesting that changes in these chromatin marks could potentially affect the modulation of *GPX7* expression.

### miRNAs potentially regulate *GPX7* expression in LGG and GBM

**Figure 7:**
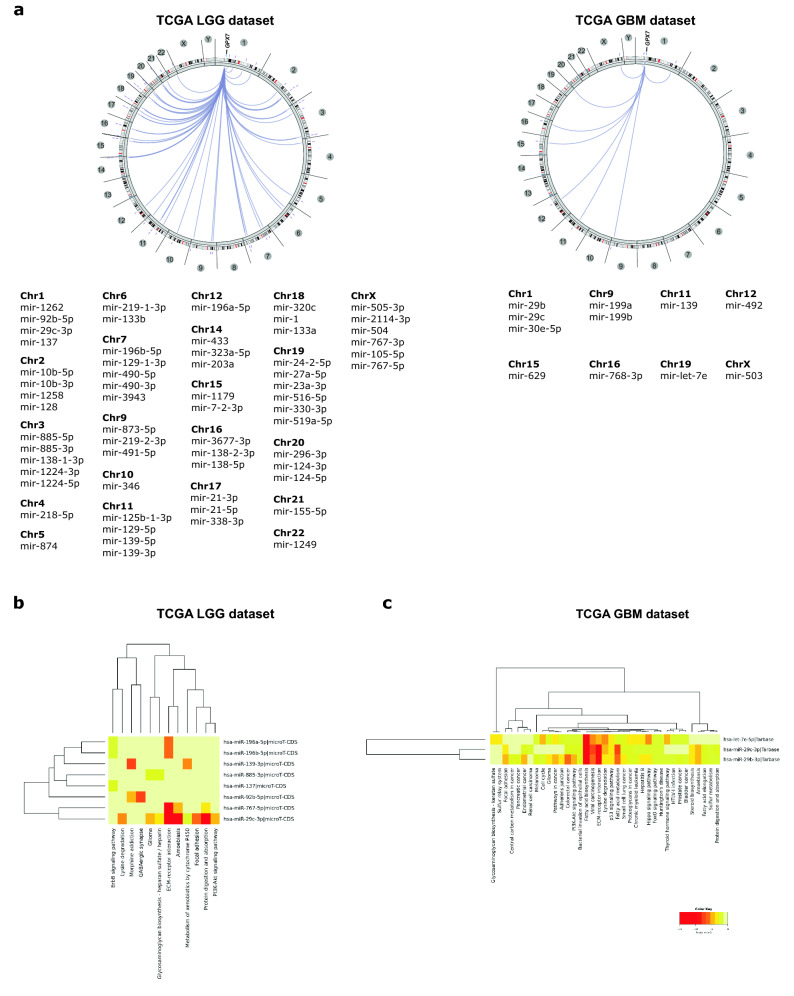
Genome-wide associations among *GPX7* and miRNAs in the LGG and GBM. (**a**) Circos plots of predicted miRNAs signatures co-expressed with *GPX7* in LGG and GBM patients from the TCGA database. Only genes with a correlation of Abs = 0.4 and *p* < 0.05 are shown in the circus plots. Chromosomes 1–Y are shown outer in the right half of the circle. miRNA genes are represented by purple lines near their chromosomal locations. The outer ring displays cytogenetic bands. In this case, lines connecting two dots represent the statistically significant correlation between two selected features: miRNA expression and *GPX7* expression. Heat-maps of significant KEGG pathways of *GPX7*-associated miRNAs by utilizing the DIANA-miRPath in (**b**) TCGA-LGG and (**c**) TCGA-GBM. Each row represents a miRNA, and each column represents the KEGG pathway^51,58–60^. Gradient from red to yellow indicates a higher to lower *p*-value (Log).

Next, to verify whether miRNAs could regulate *GPX7* expression, we identified miRNAs positively or negatively correlated with *GPX7* in LGG and GBM datasets from the TCGA (Fig. 7a, b and Supplementary Tables S5–S6). Upon identifying these miRNAs signatures in both cohorts, we focused on the gene-miRNA interactions with a primary focus on predicting the miRNAs that potentially interact with *GPX7*. A total of 8 miRNAs (hsa-mir-29c-3p; hsa-mir-137-3p; hsa-mir-767-5p; hsa-miR-196a-5p; hsa-miR-196b-5p; hsa-miR-92b-5p, hsa-miR-885-3p and hsa-miR-139-3p) were identified for LGG and 3 miRNAs (hsa-miR-29c-3p, hsa-let-7e-5p, hsa-miR-29b-3p) were identified for GBM.

To dissect the biological significance of the above selected miRNAs, DIANA-miRPath v3.0 (TargetScan database) was used to identify the signaling pathways in which these miRNAs may be involved. As shown in Fig. 7b, in LGG signature, four miRNAs (hsa-mir-29c-3p; hsa-mir-767-5p; hsa-miR-196a-5p and hsa-miR-196b-5p) prominently modulated ECM-receptor interaction, while two miRNAs (hsa-mir-29c-3p and hsa-miR-885-3p) importantly contributed to glioma development. Likewise, the miRNAs signature from GBM also affected the ECM-receptor interaction pathway (Fig. 7c). Furthermore, we also noticed a sharp correlation between all miRNAs and fatty acid biosynthesis in these tumors. These results showed that *GPX7*-associated miRNAs dysregulated different pathways in gliomas, converging mainly to the regulation of the extracellular matrix, which has a dramatic influence on glioma invasiveness and aggressiveness^91^.

### *GPX7* could be specifically upregulated in glioblastoma models treated with different therapies

Malignant primary brain tumors are a leading cause of cancer mortality in children and young adults, with few therapeutic options^92^. To date, the standard treatment for high-grade gliomas (HGGs) consists of surgical tumor resection followed by fractionated radiotherapy and chemotherapy with alkylating agents, such as temozolomide (TMZ)^93–95^, or with molecular targeted drugs (for recurrent tumors), such as bevacizumab (BEV) (an antiangiogenic drug)^96–99^ and irinotecan (a DNA topoisomerase I inhibitor)^97,100^. Furthermore, the consensus in cancer research suggests that most chemotherapeutics elevate intracellular levels of reactive oxygen species (ROS)^101^, resulting in oxidative injury and cell damage.

**Figure 8:**
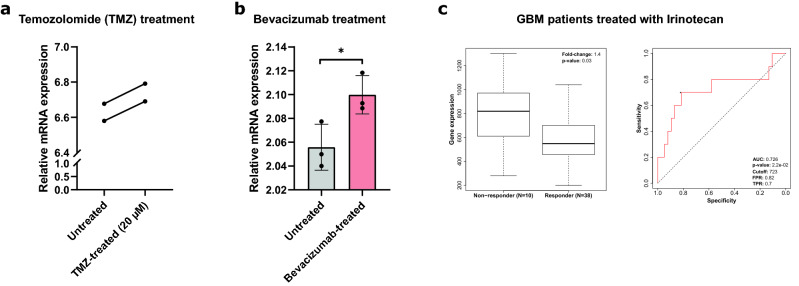
*GPX7* expression in glioma models treated with chemotherapy. (**a**) The relative *GPX7* mRNA expression of human glioblastoma cell line (U87MG) treated with temozolomide at 20 µM for 24 h (GDS4808 from GSE43452 dataset)^36^. Two-tailed Wilcoxon rank-sum test was used (*p*-value = 0.0106); (**b**) Relative *GPX7* mRNA expression in U87MG-EV human glioblastoma xenograft tumor treated with bevacizumab (10 mg/kg body weight) (GDS5672 from GSE39223 dataset)^37^. Results are shown as mean ± SD. Statistical significance is denoted by ***p < 0.001 compared with control. **p < 0.01 compared with control. *p < 0.05 compared with control; (**c**) Box plot (left) depicting the expression of *GPX7* (probe 213170_at) in GBM patients annotated with chemotherapy responses according to the Response Evaluation Criteria in Solid Tumors (RECIST) criteria. Graphs show normalized gene expression in irinotecan non-responders and responders patients. ROC plot of non-responders (N = 10) and responders (N = 23) of GBM patients treated with Irinotecan based on overall survival (OS) at 16 months. Error bar ± SD. AUC: area under the curve. PPV, positive predictive value; TPR: true positive rate.

In light of this, we proposed that different therapeutic strategies available for gliomas might affect *GPX7* expression, considering that *GPX7* acts to reduce oxidative stress^8^. To test this hypothesis, we then delved into the therapy-related microarray datasets available from the GEO database for GBM. From the GSE43452 dataset^36^, we found that the *GPX7* was significantly increased in the U87 cell line after treatment with 20 µM of TMZ for 24 h (*p* = 0.0106) (Fig. 8a). Consistent with this, we also observed a similar increase in U87-EV human GBM xenograft tumor treated with bevacizumab (10 mg/kg of body weight) when compared with non-treated tumors (GSE39223 dataset)^37^ (Fig. 8b).

Next, in order to explore the predictive potential of *GPX7* expression as a chemotherapy response predictor*,* we performed ROC (Receiver Operating Characteristic) in an independent GBM dataset (n = 454)^45^ treated with multiple chemotherapeutic agents (Supplementary Fig. S8), as described in the methods. For irinotecan treatment, the non-responder group exhibited higher expression of *GPX7* than the responder group (Fold-change = 1.4; *p* = 0.03) (Fig. 8c). Similarly, the ROC curve showed that increased *GPX7* expression could perfectly distinguish irinotecan therapy respondents from non-responders (AUC = 0.726; *p* = 2.2e–02) (Fig. 8c). Together, these findings support the idea that changes in *GPX7* expression levels are likely related to the induction of oxidative stress and ROS-mediated cell injury induced by distinct drugs approved to treat gliomas. Also, our dataset supports the application of *GPX7* expression to stratify and identify GBM patients that are likely to benefit from irinotecan treatment, given that enhanced *GPX7* expression may contribute to therapeutic resistance in GBM subpopulations.

### The biological function of co-expressed genes related to *GPX7* in LGG and GBM

**Figure 9:**
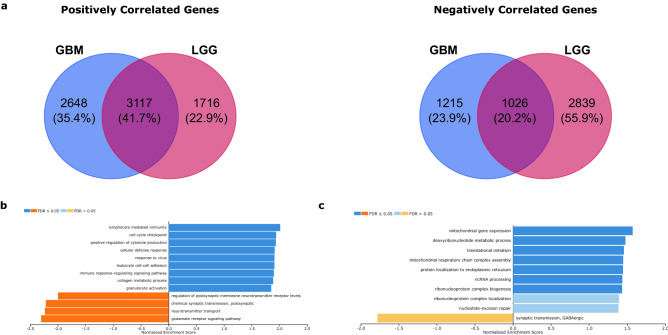
Functional enrichment analysis of *GPX7*-related genes in LGG and GBM cohorts from. TCGA. (**a**) Venn diagram of genes that positively (Left) and negatively (Right) correlated with *GPX7* expression, showing shared genes in LGG and GBM. (**b**) Functional annotation using GSEA shows an overrepresentation of Gene Ontology (GO) terms of upregulated (blue) and downregulated (orange) genes co-expressed with *GPX7* in LGG. (**c**) Functional annotation using GSEA shows an overrepresentation of Gene Ontology (GO) terms of upregulated (blue) and downregulated (orange) genes co-expressed with *GPX7* in GBM. Dark blue and orange: FDR ⩽ 0.05; Light blue and orange: FDR > 0.05. Abbreviations: FDR: false discovery rate; GO: Gene ontology.

To better understand the underlying molecular mechanisms by which *GPX7* modulates the biological processes in gliomas, we screened out the *GPX7* co-expressed gene signatures by the LinkFinder module in the LinkedOmics. In total, 4555 genes were related to *GPX7* expression (1716 positively correlated and 2838 negatively related) in LGG, whereas in GBM, we found 3863 *GPX7*-related genes (2648 positively correlated and 1215 negatively related) (Fig. 9a). Using gene set enrichment analysis (GSEA), we observed that the GO biological process profiles were markedly different in both cohorts. In LGG, GO terms mainly fit into immune mechanisms involving both innate and adaptive immunity, type I interferon production and regulation of synaptic transmission (Fig. 9b). By contrast, marked upregulation of the metabolic regulation of mitochondrial dynamics, translation and ribosomal synthesis signature along with downregulation of a synaptic transmission signature was seen in GBM (Fig. 9c). Our data here provide a basis to explore clinically relevant biological processes involved in gliomagenesis that can be used as potential targets in translational medicine.

### *GPX7* expression is associated with immune cell infiltration

**Figure 10:**
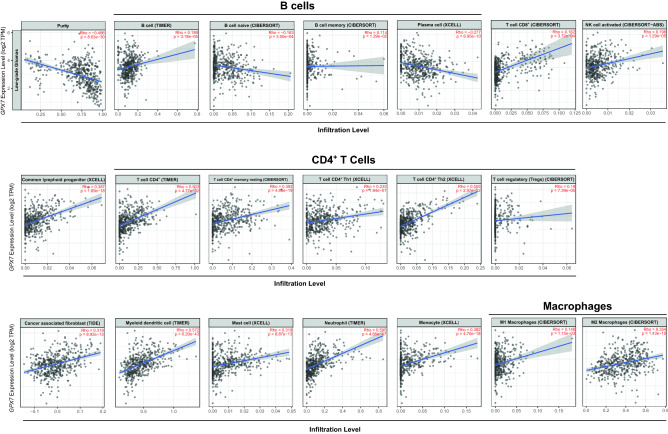
Correlation of *GPX7* expression with immune infiltration level in LGG (TCGA dataset) via Tumor Immune Estimation Resource (TIMER) database. All spearman correlations were adjusted for tumor purity.

**Figure 11:**
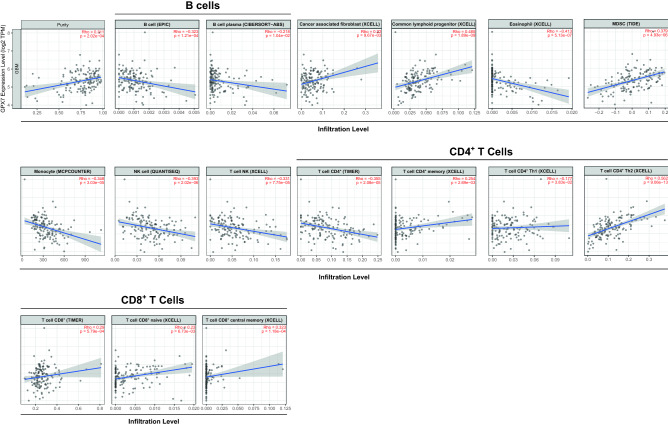
Correlation of *GPX7* expression with immune infiltration level in GBM (TCGA dataset) via Tumor Immune Estimation Resource (TIMER) database. All spearman correlations were adjusted for tumor purity.

Given the heterogeneity of the tumor immune microenvironment, the close relationship between immunological features and the therapeutic response in gliomas^102,103^, and the association found between *GPX7*-co-expressed genes with immune-related gene ontology terms, especially in LGG, we reasoned that *GPX7* expression could have potential to impact the immune cell infiltration. Here, we found that *GPX7* was negatively associated with the tumor purity in LGG (*ρ* = − 0.486; *p* = 8.63e−30) (Fig. 10 and Supplementary Table S7) but positively correlated to it in GBM (*ρ* = 0.311; *p* = 2.02e−04) (Fig. 11 and Supplementary Table S7). These results are in accordance with the finding of gene ontology terms for *GPX7*-co-expressed genes being related to stromal reactions (e.g.: immune cell infiltration and IFN-I response) in LGG, but being mainly related to metabolic processes in GBM, suggesting that while in LGG *GPX7* activation directly impacts the tumor microenvironment, in GBM the affected processes are tumor-cell intrinsic.

Moreover, in LGG, *GPX7* expression was positively correlated with multiple immune-stromal cells, such as B cells (*ρ* = 0.189; *p* = 3.18e−05), common lymphoid progenitors (*ρ* = 0.387; *p* = 1.69e−18), monocytes (*ρ* = 0.382; *p* = 4.70e−18), M1 (*ρ* = 0.148, *p* = 0.001) and M2 macrophages (*ρ* = 0.354, *p* = 1.42e−15), mast cells (*ρ* = 0.319; *p* = 8.87e−13), myeloid dendritic cells (*ρ* = 0.572; *p* = 6.20e−43), neutrophils (*ρ* = 0.594; *p* = 4.65e−47), CD4 + T cells (LGG, *ρ* = 0.503; *p* = 4.77e−32), Treg cells (*ρ* = 0.180; *p* = 7.39e−05), CD8^+^ T cells (*ρ* = 0.162; *p* = 0.0003), NK cells (*ρ* = 0.198; *p* = 1.29e−05) and cancer-associated fibroblasts (CAFs, *ρ* = 0.319; *p* = 8.92e−13) (Fig. 10; Supplementary Table S7). Furthermore, after purity-related adjustment, we found that *GPX7* expression was related to high intratumor expression of most markers of T cells (general) (*CD3E, CD3D, CD2*), B cells (*CD19, CD79A*), monocytes (*CD86, CD115*), TAMs (*CCL2, CD68, IL10*), M2 Macrophages (*CD163, VSIG4, MS4A4A*), neutrophils (*CD11b, CCR7*), dendritic cells (*HLA-DPB1, HLA-DQB1, HLA-DRA, HLA-DPA1, BDCA-1, BDCA-4*), CD4^+^ T cells (*CD4, CD45RA*), Th1 cells (*T-bet, STAT4, STAT1, IFN-g, TNF-a*), Th2 cells (*GATA3, STAT6, STAT5A*) and Treg cells (*FOXP3, CCR8, STAT5B, TGFβ*) (Supplementary Table S8). Thus, these data indicated that high *GPX7* expression is associated with a highly inflammatory microenvironment and immune exhaustion in these tumors.

Otherwise, in GBM, *GPX7* only positively correlated with common lymphoid progenitors (*ρ* = 0.485; *p* = 1.89e−09), CD8^+^ (*ρ* = 0.290; *p* = 0.0005), myeloid-derived suppressor cells (*ρ* = 0.379; *p* = 4.93e−06) and CAFs (*ρ* = 0.220; *p* = 0.009), while it was negatively correlated with B cells (*ρ* = − 0.322; *p* = 0.0001), eosinophils (*ρ* = − 0.413; *p* = 5.13e−07), monocytes (*ρ* = − 0.348; *p* = 3.03e−05), CD4 + T cells (*ρ* = − 0.354; *p* = 2.08e−05), NK cells (*ρ* = − 0.393; *p* = 2.02e−06) and NKT cells (*ρ* = − 0.331; *p* = 0.0006) (Fig. 11 and Supplementary Table S7). Besides, exploring the correlation between *GPX7* and sets of immunological markers using the TIMER2.0 database, we also observed significant correlations between *GPX7* and the monocyte marker *CD115*, the M1 macrophage marker *IRF5*, the neutrophil marker *CD11b*, the NK marker *KIR2DL3*, CD4^+^ marker *CD11c*, the Th2 marker *STAT6*, the Tfh marker *BCL6*, the Th17 marker *STAT3* and Treg markers *FOXP3, STAT5B, TGFβ* (Supplementary Table S8), therefore suggesting that *GPX7* expression differently affect tumor immune-stroma in these types of cancer.

**Figure 12:**
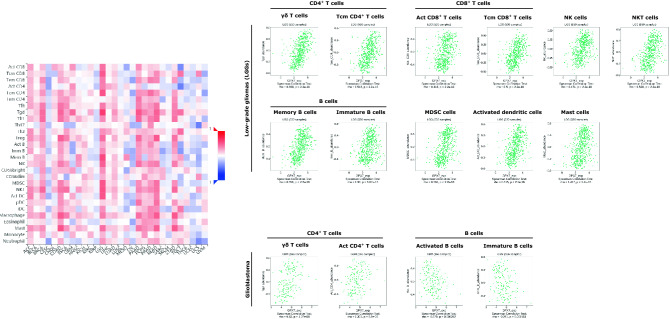
Correlation between *GPX7* expression and tumor-infiltrating immune cells. Left panel: Correlative heat-map between *GPX7* and 28 tumor-associated immune cells (Vertical axis) in a pan-cancer cohort (Horizontal axis). The colors indicate the correlation coefficients; Upper panel: correlation between *GPX7* expression and immune-related signatures from (i) gamma delta T cells **(**γδ T cells); (ii) central memory CD4^+^ T cells (Tcm CD4^+^ T cells); (iii) activated CD8^+^ T cells (Act CD8^+^ T cells); (iv) central memory CD8^+^ T cells (Tcm CD8^+^ T cells); (v) NK cells; (vi) NKT cells; (vii) memory B cells; (viii) immature B cells; (ix) Myeloid-derived suppressor cells (MDSC cells); (x) activated dendritic cells; (xi) mast cells in low-grade gliomas (LGG). Lower panel: correlation between *GPX7* expression and immune-related signatures from (i) gamma delta T cells **(**γδ T cells); (ii) activated CD4^+^ T cells (Act CD4^+^ T cells); (iii) activated B cells; (iv) immature B cells in glioblastoma (GBM).

Consistently, closer inspection of immune-related signatures from the TISIDB database revealed that a variety of subtypes of tumor-infiltrating lymphocytes (TILs) were significantly affected by *GPX7* expression in LGG including γδ T cells, conventional CD4^+^ T cells (memory CD4^+^ T cells—Tcm CD4), CD8^+^ T cells (Activated and Central memory CD8^+^ T cells), B cells (memory and immature B cells), NK cells, NKT cells, myeloid-derived suppressor cell (MDSC), activated dendritic cell, and mast cell. Differently, in GBM, *GPX7* only showed a close connection with γδ T cells, activated CD4^+^ T cells, and activated and immature B cells (Fig. 12). Based on these results, it is conceivable that the correlation of immune cells and *GPX7* expression may be characteristic of tumor/tissue type, which emphasizes the uniqueness of immune infiltration in gliomas.

The burgeoning field of immune cell migration into solid tumors has created new and exciting opportunities in translational cancer immunotherapy^104^. In this context, it is now clear that the migration of both effector and suppressive immune cell types to the tumor microenvironment (TME) is controlled by a plethora of chemokines. As we found immune cells to be potentially influenced by *GPX7*, we next hypothesized that *GPX7* could be acting to modulate immune cell migration. To test this, we compared *GXP7* expression with key chemokines and their corresponding receptors. As shown in Supplementary Table S9, the most relevant chemokines correlated with *GPX7* were: (i) in LGG, CCL1/2/5/20/22/25 and CXCL9/10/11/16; (ii) and in GBM, CCL4/16 and CXCL1. Concerning the chemokine receptors, CCR1/2/5, CXCR2/3/4/6, XCR1 and CX3CR1 showed the greatest correlation with *GPX7* in LGG, while CCR1/5, CXCR2 and CX3R1 were the most significantly correlated in GBM.

To gain a better understanding of the modulation of TILs migration into the LGG TME, further associations between *GPX7* and immunomodulators, including immune checkpoint receptors, activating receptors and MHC molecules, were examined. We identified striking positive correlations with most immune checkpoints and immune suppressive receptors in LGG, such as *TIM3* (*HAVCR2*), *IL10RB* (interleukin 10 receptor subunit beta), *LGALS9* (galectin 9), *PDCD1LG2* (programmed cell death 1 ligand 2), *TGFB1* (transforming growth factor beta 1) and *TGFBR1* (transforming growth factor beta receptor 1). In contrast, only *IL10RB* was highly associated in GBM (Supplementary Fig. S9). Amongst the immunostimulators, *CD276*, *CD40*, *CD48*, *CD86*, *IL6*, *MICB*, *TMEM173* and *TNFRSF8* presented the highest correlation coefficients in LGG, whereas *TNFRSF25, IL6R* displayed the highest correlation coefficients in GBM (Supplementary Fig. S10). Moreover, almost all MHC‐related genes were highly correlated with *GPX7* expression in LGG, but weakly correlated in GBM (Supplementary Fig. S11).

**Figure 13:**
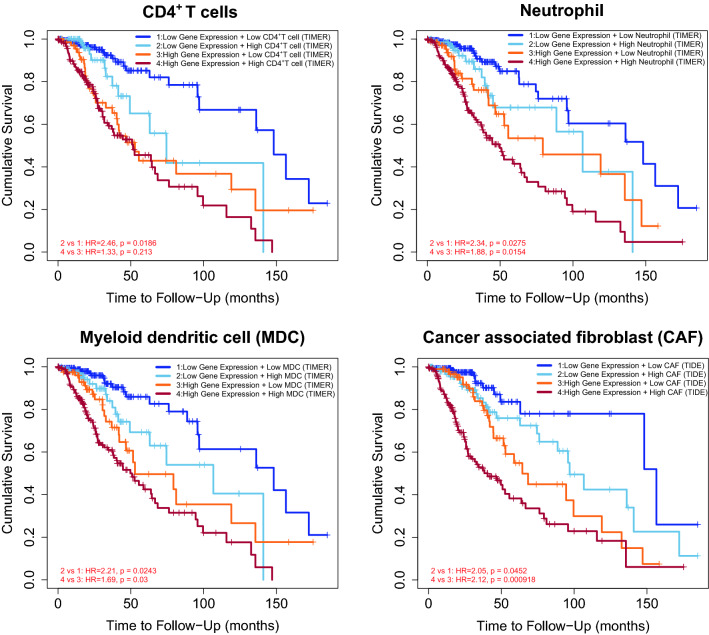
Effect of *GPX7* expression and immune infiltration on TCGA-LGG patients overall survival (OS). Kaplan–Meier plot displaying differences in OS among patients stratified by both the predicted infiltration level of immune cells (CD4^+^ T cells, neutrophil, myeloid dendritic cell and cancer-associated fibroblast) and *GPX7* expression level in LGG. High and low immune infiltration cutoffs were determined based on the infiltration score greater than or less than 0, while high and low *GPX7* expression cutoffs were defined using the median. The prognostic signature built by the immune infiltration of CD4^+^ T cells, neutrophil, myeloid dendritic cell and *GPX7* expression was profiled by TIMER algorithm, while the signature of cancer associated fibroblast and *GPX7* expression was profiled by TIDE. P-values were calculated using the log-rank test and vertical hash marks indicate censored data.

Finally, we investigated the relationship between each immune cell type and overall survival (OS) in LGG and GBM patients. All patients were initially divided into high and low-expressing groups according to the TILs and *GPX7* expression. Interestingly, the survival curves showed that low expression of *GPX7* along with low immune infiltration of CD4^+^ T cells, neutrophil, myeloid dendritic cell (mDCS) and cancer associated fibroblast (CAF) have better prognosis than the high TIL- and *GPX7*-expressing group in LGG (Fig. 13). In contrast, no association with survival was observed in GBM.

Together, these findings strongly suggest that *GPX7* may be involved in tumor inflammatory response, which might be responsible for modulating the immune molecules and TILs migration, impacting patient outcomes, especially those with LGG.

## Discussion

Gliomas are characterized by a complex TME which plays a critical role in tumor invasiveness, malignancy and therapy failure. Current evidence suggests that the reactive oxygen species (ROS) derived from redox (reduction–oxidation) imbalance in the TME may contribute to genomic damage, promoting DNA mutations and activating oncogenes^105^, and also affect intracellular signal transduction of multiple cellular pathways, thus conferring specific conditions for initiation and malignant progression of many human tumors, including gliomas^105–109^. As part of the antioxidant system in mammals, the GPXs family is a major regulator of cellular redox state^7,8^. In particular, the non-selenocysteine-containing *GPX7* has been shown to be essential for the maintenance of redox homeostasis through oxidative protein folding^5–7^ and releasing of the non-targeting short interfering RNAs (siRNAs)-associated stress^8,9^, by converting superoxide to water. Despite the controversial role of *GPX7* in carcinogenesis^5,23^, there is limited literature on the potential mechanisms of *GPX7* expression in gliomas. Herein, our findings provide insights into understanding the epigenetic regulation and the potential association of *GPX7* with clinical features and immunity of gliomas.

In our study, we firstly found up-regulation of *GPX7* in a pan-cancer analysis, suggesting that *GPX7* might function as an extensive tumor-promoter^23,110^. Further interrogating glioma tumors from multiple cohorts, we found that this peroxidase was markedly higher as pathological grade increased. Its expression was also strongly impacted by other clinicopathological and molecular characteristics, which conferred a worse outcome. These results, along with a recent report^111^ provide firm evidence of the oncogenic role of *GPX7* in gliomas, pointing out its crucial activity in tumor progression and survival.

There is a growing awareness that *GPX7* deficiency increases sensitivity to oxidative stress. However, the pathways regulating this process remain elusive, especially in the context of gliomas. Although *GPX7* depletion is non-lethal, *GPx7*^−/−^ mice carry multi-organ abnormalities (e.g. cardiomegaly, splenomegaly, glomerulonephritis and fatty liver) due to increased systemic oxidative stress damage, in addition to increased risk of carcinogenesis, severe oxidative DNA damage and reduced lifespan^10^. Similarly, it is also known that *GPx7*-deficient cells accumulate endogenous ROS, lowering cellular viability^8,10^, and especially in breast cancer cells, *GPX7* is essential for reducing the oxidative stress generated by specific polyunsaturated fatty acids^112^. Furthermore, we now have evidence that mutations in *TP53* and *EGFR* attenuate ROS accumulation in cancer cells by sustaining an oncogenic oxidant intracellular environment through an integrated regulation of redox-related enzymes and signaling pathways (e.g. PI3K-AKT signaling, MAPK cascade, ERK and the redox-sensitive IκK/NF-κB pathway)^113,114^, thereby supporting proliferation, protein synthesis and invasion^106,115–118^. In line with this, we demonstrated that *GPX7* was overexpressed in glioma cell lines in a cell cycle-dependent manner and was higher in patients harboring mutations in *TP53* and *EGFR*, suggesting its involvement in cancer cell growth associated with a response to a progressive accumulation of ROS, probably driven by mutant *TP53* and *EGFR* combined with a metabolic reconfiguration and a coordinated regulation of other factors present in the glioma TME (e.g. hypoxia, inflammatory cytokines, growth factors)^109,119^.

In addition, *GPX7* expression increased in chemotherapy-resistant GBM patients, supporting the idea that changes in *GPX7* expression are likely to be related to the induction of oxidative stress and ROS-mediated cell injury induced by specific drugs approved to treat gliomas. The results of many studies support a scenario in which increased ROS levels control the multidrug resistance of cancer cells in multiple ways, which leads to development and metastasis during or after chemotherapy. Such resistance is the result of a process known as “Redox Resetting”^120,121^, where a new redox balance is established with a higher ROS level through the upregulation of the ROS-scavenging system, such as *GPX7*, which can confer drug resistance^118,122–124^. Although our results suggest the importance of *GPX7* in relieving oxidative stress in gliomas, it becomes of paramount importance to reveal the molecular mechanisms underlying the redox status in this type of cancer, since they might potentially open the discovery of appropriate strategies to selectively modulate ROS for the “oxidation therapy”.

Tumorigenesis is due to the combined action of multiple epigenetic events, such as DNA methylation, histone modifications, chromatin remodeling and microRNAs (miRNAs)^125^. In light of this evidence, it is not surprising that these epigenetics processes have been reported to be involved with every aspect of pathophysiology, diagnosis, and treatment of gliomas^126–128^. However, the epigenetic mechanisms that potentially modulate *GPX7* expression in gliomas are still unknown. Our analysis established that DNA hypomethylation in the CpG islands and shores may be one of the mechanisms in leading to *GPX7* overexpression in LGG, agreeing with Peng et al.^21^ and Chen et al.^22^, who also reported the same negative correlation when analyzing the promoter region of the *GPX7* in esophageal adenocarcinomas and gastric cancer, respectively. Considering that ROS impacts the activities of epigenetic modulators^129–131^, it is reasonable to hypothesize that high levels of ROS generated by increased metabolic rate, gene mutation and relative hypoxia^117^ perhaps explain the DNA hypomethylation and *GPX7* overexpression seen in gliomas. This is presumably due in part to ROS-induced oxidation of adenine (8-oxo-A) and guanine (8-oxo-G), which impairs DNA methylation patterns, since damaged bases in the nascent DNA strand can either inhibit the methylation of cytosine within a distance of 1–2 bp (base pairs) or the binding to the methyltransferase, thereby leading to global hypomethylation^132–135^.

Moving beyond the DNA methylation regulation, we found a close correlation between *GPX7* expression with the deposition of active histone modifications, which may support a crosstalk model between DNA hypomethylation and the H3K9ac and H3K27ac deposition at active regulatory elements and at the TSS region, which establish a chromatin conformation that is compatible with *GPX7* expression^136^. Indeed, it has been shown that in cancer cells, the accumulation and spreading of H3K27ac enhance oncogene expression, and the chromosomal rearrangements and genetic alterations of HAT activity can also influence the frequency of interactions of these chromatin structures, thereby favoring gene expression^137^. Accordingly, DNA hypomethylation and/or reduced H3K27me3 point to be the major driving forces of activated gene expression in K27M mutant pediatric high-grade gliomas (pHGGs)^138^. As such, H3.3K27M mutation accompanied by increased total H3K27ac and reduction in H3K27me3, leading to glioma formation and subsequent tumor progression in a brainstem glioma model^139^. Lastly, another potential insight into the epigenetic mechanisms related to *GPX7* expression comes from our co-expression analyses showing that specific miRNA signatures can regulate *GPX7* in gliomas, concurrent with a previous report wherein miR-137 and miR-29b were shown to bind to the 3′ UTR region of *GPX7* and inhibit its expression in both SW480 (human colon adenocarcinoma) and HEK293 (human embryonic kidney cell line) cell lines^140^. Thus, the functional relationship between *GPX7* expression and epigenetic modifications is complex and further mechanistic studies are required to explain this dependency.

We next explored the genes significantly associated with *GPX7* and their functions in gliomas. Conceivably, our data indicated that the functions of *GPX7* and associated genes were primarily involved in immune mechanisms in LGG. In contrast, in GBM, they were related to the metabolic regulation of mitochondrial dynamics. Curiously, a recent pathway-based classification suggested that GBM tumors classified as mitochondrial exhibited marked vulnerability to inhibitors of oxidative phosphorylation (OXPHOS), which increased intracellular ROS and sensitivity to radiotherapy, thus strengthening the potential application of synergic *GPX7* and OXPHOS inhibition in mitochondrial subtype to boost antitumor and immune responses^141^.

Importantly, this study consistently showed that *GPX7* expression has a dramatic association with immune infiltration and the degree of activation of diverse immune cells using different algorithms. Furthermore, this association was tumor-type dependent, especially prominent in LGG but not in GBM. Interestingly, *GPX7* expression and other enzymes associated with redox balance were also increased in other inflammatory contexts, such as chronic HCV infection^20^, suggesting that this pathway may be upregulated as a feedback mechanism in these contexts to cope with oxidative stress generated in these conditions. Therefore, upregulation of *GPX7* observed in gliomas might reflect the oxidative stress that these tumors experience during their development, as supported by its positive correlation with the cell cycle, chemotherapy treatment, and inflammatory pathways and cells. Prominent among these cells, neutrophils, CD4^+^ T cells, mDCs and CAFs, along with the *GPX7* expression, exerted a significant negative influence on the OS of LGG, suggesting once again that both *GPX7* and immune cell infiltration increase along with tumor aggressiveness, probably driven by increased oxidative stress in the tumor microenvironment. Of note, GBMs are known to be poorly immunogenic due to active production of immunomodulatory molecules by tumor cells^142^, which conceivably explains the lack of association between *GPX7* and inflammation in these tumors.

Most glioma patients have a strong neutrophilia^143,144^, and neutrophil infiltration at the tumor site has been associated with decreased overall survival (OS), tumor recurrence in grade 2–4 glioma patients and brain metastasis^145^. Circulating neutrophils are recruited at the tumor site by CXCL8 produced by FasL triggering on glioma cells^146,147^ or by the Migration Inhibitory Factor (MIF) produced by glioma cancer stem cells^148^. At the tumoral site, tumor-associated neutrophils (TANs) support glioma infiltration and progression, respectively, secreting elastase^149^ and neutrophil extracellular traps (NETs)^150^, which regulate the HMGB1/RAGE/IL-8 axis^150^, that ultimately leads to ROS generation, thus partially explaining, therefore, the upregulation of *GPX7* associated with neutrophil infiltration seen in our study. Additionally, our results align well with the involvement of CCL2/5, CCR2, and CXCR2/4 in regulating the TAN homing to glioma microenvironment, as we identified striking positive correlations of *GPX7* with these chemokines/receptors^151–153^, thus revealing the potential role of *GPX7* in the mobilization and recruitment of TANs through this pathway.

The role of CD4^+^ T lymphocytes is increasingly being studied in the T-cell response against tumors. High infiltration of CD4^+^ T cells is generally considered to be associated with angiogenesis promotion^154^, recurrence/progression^154^, and unfavorable prognosis of gliomas^154,155^. Similarly, our study highlights that LGG patients with high *GPX7* expression and increased frequency of CD4^+^ T cells have worse OS. Together with the extensive epigenetic reprogramming dictated by the glioma TME, the prominent association between *GPX7* expression and CD4^+^ cell subtype signatures (e.g., Th1, Th2 and Treg) seen in our study suggests the role of *GPX7* in CD4^+^ T cell polarization, which may participate in the lack of effective immune activation against gliomas^156,157^. The results mentioned above, along with the strong association between *GPX7* and several immunosuppressive cells (e.g., NKT cells, TAMs, and MDSCs) or inhibitory immune checkpoint markers (e.g., *PD-1, CTLA-4, LAG-3, TIGIT, TIM-3* and *CD96*) further enhances the importance of *GPX7* in the regulation of an immunosuppressive tumor microenvironment^158–162^.

Several studies have highlighted the suppressive impact of *TIGIT* on a wide range of immune functions and immune cells (e.g., T/NK cells)^163–165^. Besides, TIGIT^+^ Treg cells selectively inhibit pro-inflammatory Th1 and Th17 cell responses, but not Th2 cell responses^166^. Interleukin 4 (IL-4) produced by Th2 cells promotes the differentiation of TAM to M2 macrophages, leading to an immunosuppressive phenotype. Interestingly, there is some circumstantial evidence that M2 macrophages require ROS from TME for proper polarization and acquisition of the pro-tumorigenic phenotype^167^. ROS induces its polarization via IL4-induced Stat3 activation^167^, up-regulation of PD-L1, mediated by the NF-κB, secretion of immunosuppressive cytokines^168^, and the differential expression of ROS scavenging enzymes, such as GPx. Our data report a strong correlation between *GPX7* expression with the M2 macrophages markers (*CD163, VSIG4, MS4A4A*), implying a potential role for *GPX7* in the polarization of tumor-associated macrophages (TAM) in LGG^169^.

mDCs are essential players in coordinating the activation of both CD4^+^ and CD8^+^ T cells and initiate adaptive immune responses in infectious contexts by capturing and presenting antigens from inflamed tissues to T cells in the lymph nodes^170^. However, a failure in mDC activation in microenvironments dominated by immunomodulatory molecules (e.g., TGFβ and IL-10) and cells (such as Tregs) can promote a mDC tolerogenic phenotype, inducing T cell anergy upon antigen presentation, which is a frequent process in cancer, especially in well-known cold tumors with decreased mutational burden^171^, as is the case for gliomas^172^. Finally, cancer-associated fibroblasts (CAFs) can be activated by ROS and inflammation in the tumor microenvironment^173^ and are also consistently implicated in tumor progression by supporting tumor growth and invasion through secretion of cytokines and extracellular matrix remodeling components^174^. Among the CAF-derived cytokines, TGFβ can be highlighted by its effects in inducing EMT and maintenance of stemness in cancer cells, as well as by its immunosuppressive roles^175,176^. Interestingly, CAFs were shown to induce a suppressive phenotype in dendritic cells that was dependent on ROS generation in the lung cancer microenvironment, suggesting a mechanism that connects CAFs, mDCs and ROS to cancer immunosuppression and progression^177^.

In light of these data, it is reasonable to suggest that inflammation and ROS generation are interconnected in a feedforward loop during tumor development, triggering *GPX7* expression. Notably, the negative impact of neutrophils, mDCs, CD4^+^ T cells and CAFs associated with increased *GPX7* in LGG prognosis suggests that ROS-associated inflammation and immune cell infiltration in these cancers are associated with tumor progression rather than elimination, possibly due to immunosuppressive mechanisms operating in the TME, which might also be induced by oxidative stress. The biology of *GPX7* and its effects in multiple cell types and pathologic contexts are only beginning to be dissected, and further mechanistic studies investigating *GPX7* roles in cancer and immunity might reveal more complex mechanisms.

In conclusion, we have shown that *GPX7* overexpression has an oncogenic role and was related to worse clinical evolution in gliomas. Importantly, we showed that this increase was associated with greater histological or molecular features of glioma malignancy, and we suggest that this upregulation might be related to a progressive accumulation of ROS in the TME. Notably, we provided the first evidence regarding the epigenetic-mediated regulatory mechanisms underlying *GPX7* activation in gliomas. Furthermore, our study yields critical insights into the significant effect of *GPX7* in modulating the immune molecules and immune cell infiltration in the microenvironment of gliomas, impacting patient outcomes, opening up future opportunities to regulate the immune response.

## Supplementary Information

Supplementary Information.

## Data Availability

The datasets analyzed for this study can be found in the Chinese Glioma Genome Atlas (CGGA) (http://www.cgga.org.cn/), GEPIA (http://gepia.cancer-pku.cn/index.html), GlioVis (http://gliovis.bioinfo.cnio.es/), Oncomine (http://www.oncomine.org), TISIDB (http://cis.hku.hk/TISIDB), Tumor Immune Estimation Resource (TIMER, https://cistrome.shinyapps.io/timer), TCGA databases (https://tcga-data.nci.nih.gov/tcga/), Cancer Cell Line Encyclopedia (CCLE, https://portals.broadinstitute.org/ccle/), The Gene Expression Omnibus (GEO) (https://www.ncbi.nlm.nih.gov/gds) and University of California Santa Cruz (UCSC) Xena Browser (https://xena.ucsc.edu/). Further inquiries can be directed to the corresponding author.

## Abbreviations

BEV: Bevacizumab
CNA: Copy number alteration
CGGA: Chinese Glioma Genome Atlas
DFS: Disease-free survival
GBM: Glioblastoma
*GPX7*: Glutathione peroxidase 7
HAT: Histone acetyltransferase
HGGs: High-grade gliomas
LGG: Low-grade gliomas
OS: Overall survival
ROS: Reactive oxygen species
TCGA: The Cancer Genome Atlas
TME: Tumor microenvironment
TMZ: Temozolomide
