# Supplementary material for: Comprehensive analysis of epigenetics regulation, prognostic and the correlation with immune infiltrates of GPX7 in adult gliomas
Source: Sci Rep. 2022 Apr 19;12:6442. doi: 10.1038/s41598-022-10114-1 (PMC9018725; doi:10.1038/s41598-022-10114-1)
Supplement: Supplementary file 1 — Supplementary Information. [file 41598_2022_10114_MOESM1_ESM.docx]

**Comprehensive analysis of epigenetics regulation, prognostic and the correlation with immune infiltrates of *GPX7* in adult gliomas**

Wallax Augusto Silva Ferreira^1,*^, Glauco Akelinghton Freire Vitiello^2^, Tiago da Silva Medina^2,3^, Edivaldo Herculano Correa de Oliveira^1,4^.

**Supplementary Information**


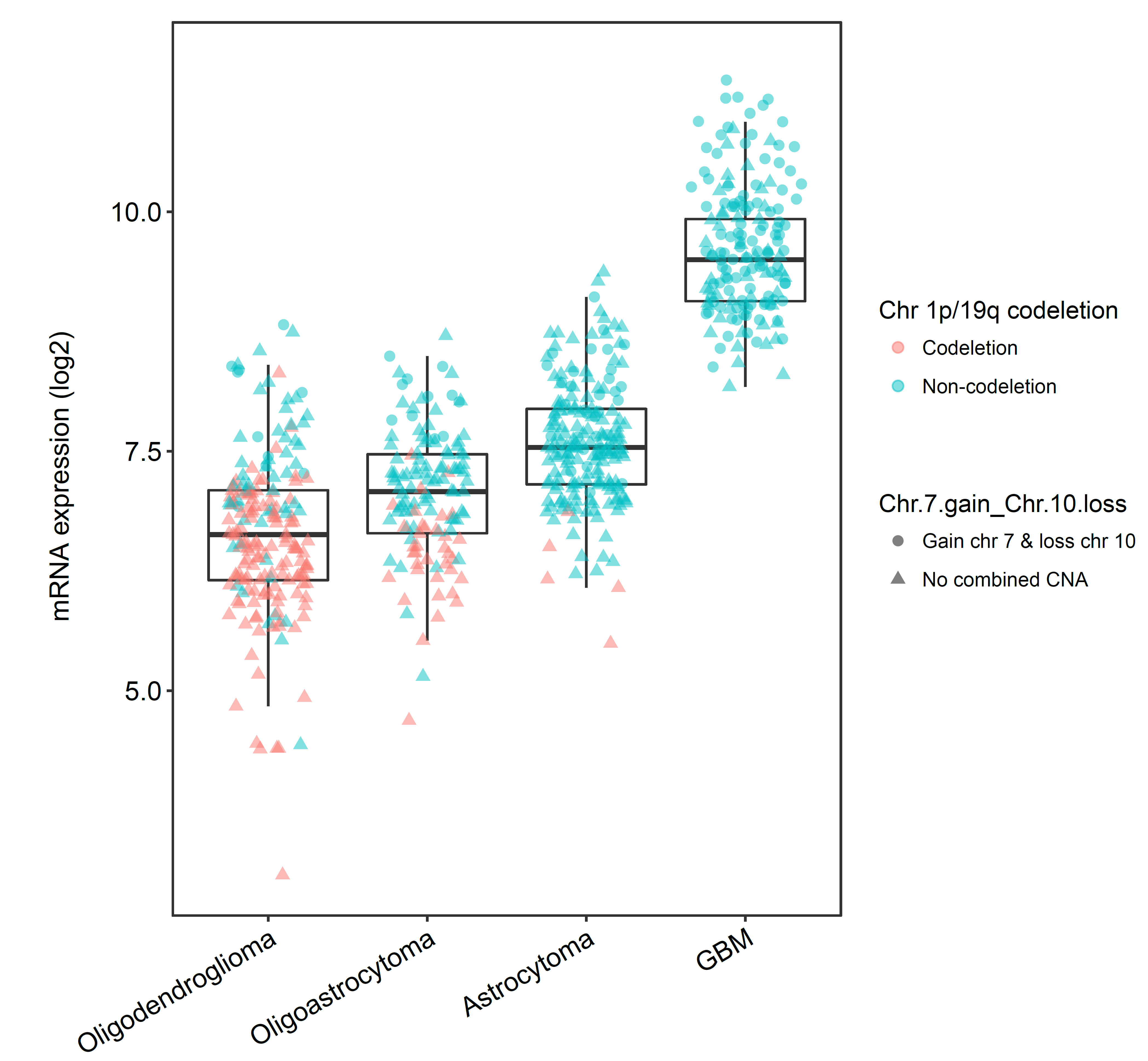
**Supplementary Figures**

**Supplementary Figure S1:** *GPX7* mRNA expression in different gliomas from TCGA according to 1p/19q codeletion and concomitant gain of chr7/loss chr10.


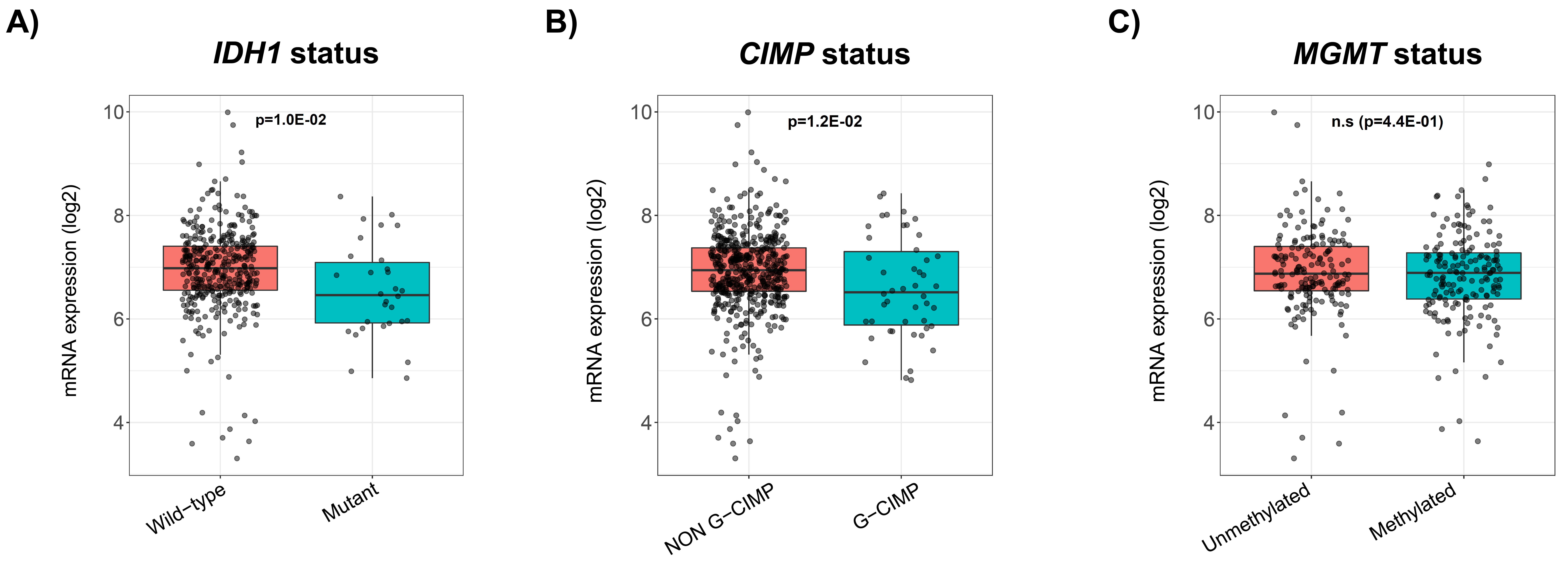
**Supplementary Figure S2:** *GPX7* expression in the GBM cohort (TCGA) according to **(a)** *IDH1* promoter mutation status, **(b)** *CIMP* status, and **(c)** *MGMT* methylation status.


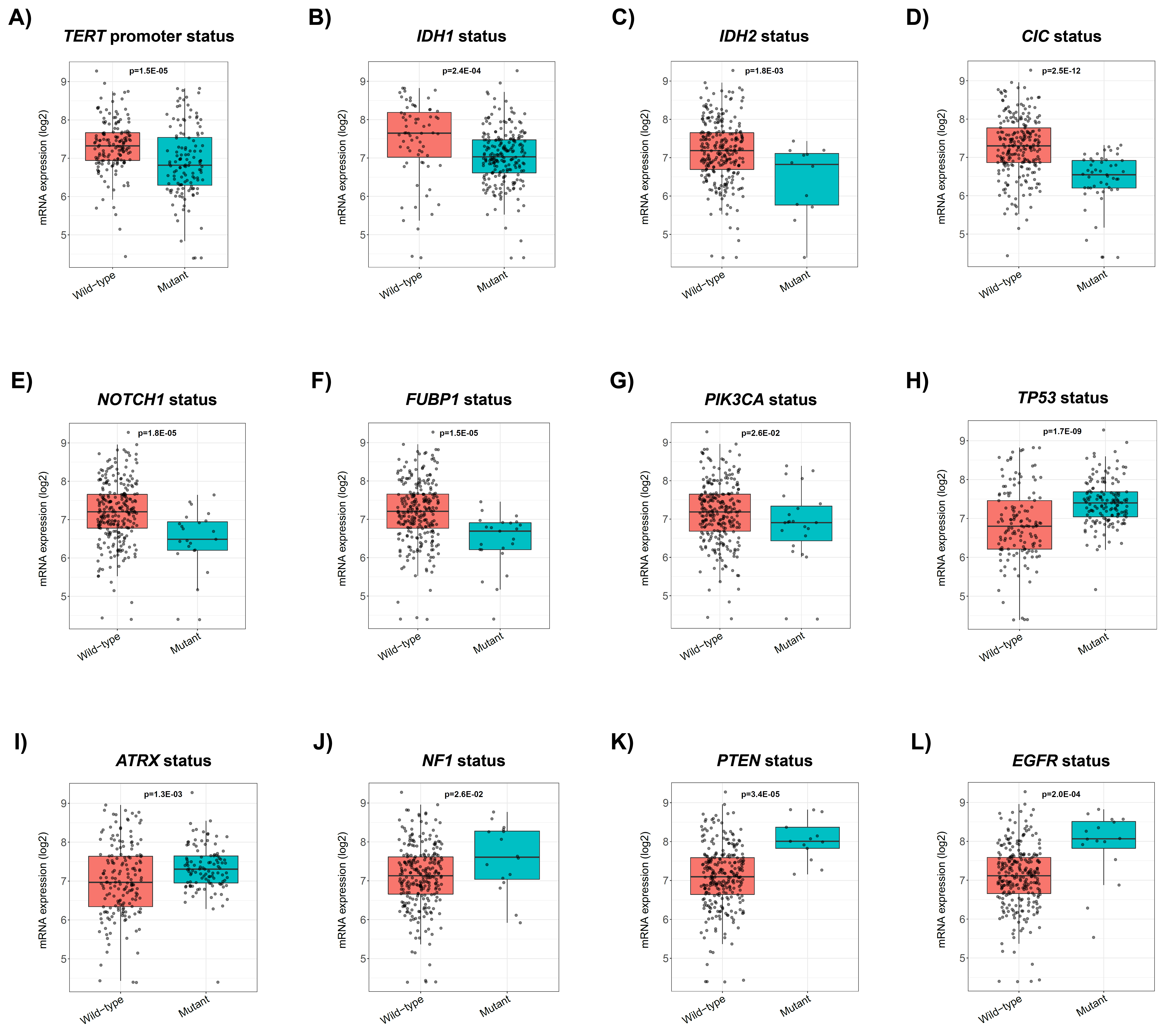
**Supplementary Figure S3:** *GPX7* expression concerning the most mutated genes in the LGG cohort (TCGA). **(a)** *TERT* promoter mutation status; **(b)** *IDH1* promoter mutation status; **(c)** *IDH2* promoter mutation status; **(d)** *CIC* promoter mutation status; **(e)** *NOTCH1* promoter mutation status; **(f)** *FUBP1* promoter mutation status; **(g)** *PIK3CA* promoter mutation status; **(h)** *TP53* promoter mutation status; **(i)** *ATRX* promoter mutation status; **(j)** *NF1* promoter mutation status; **(k)** *PTEN* promoter mutation status; **(l)** *EGFR* promoter mutation status.


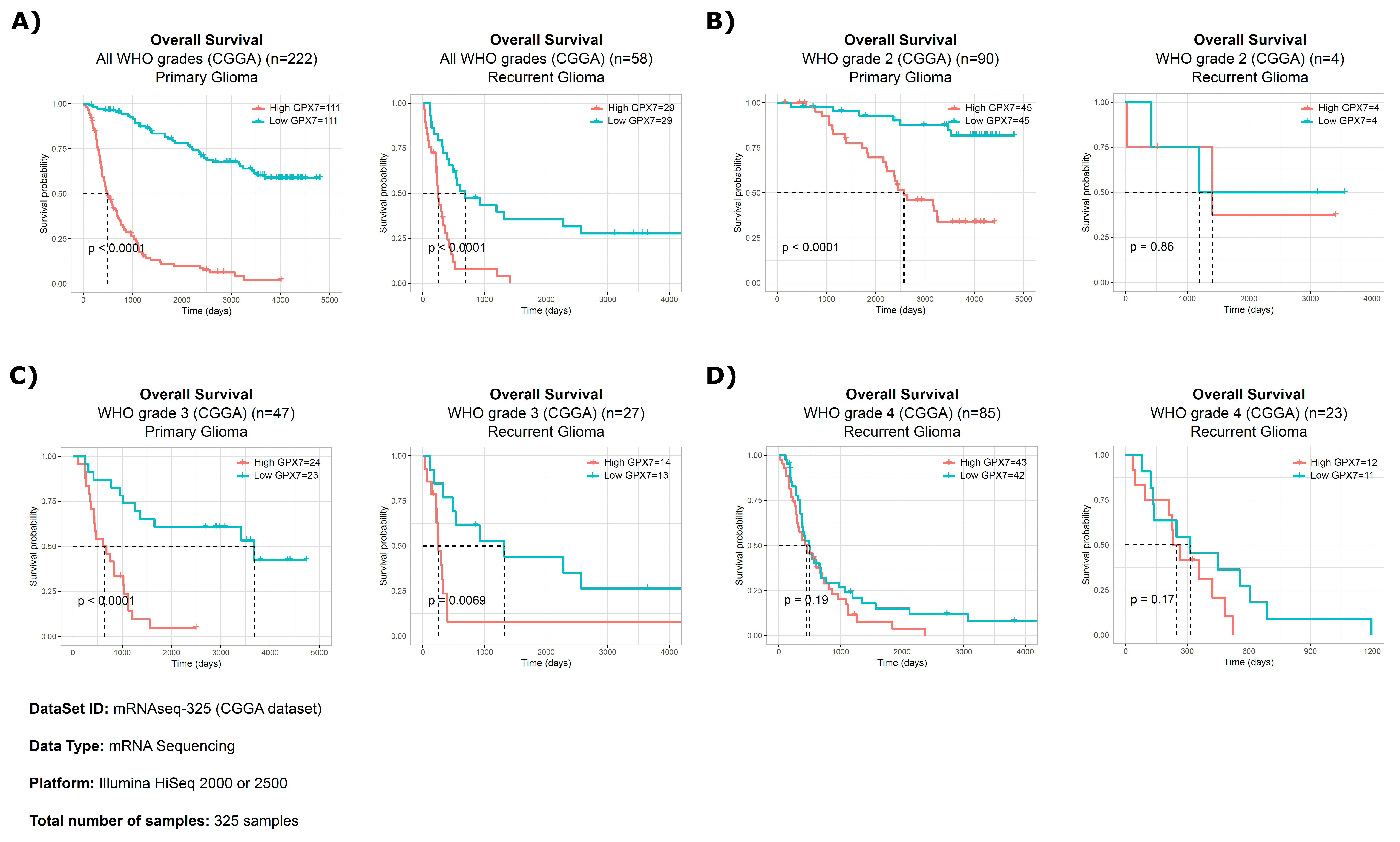
**Supplementary Figure S4:** Kaplan-Meier survival curves based on high (red) and low (green) expression of *GPX7* in gliomas from the CGGA database (Chinese Glioma Genome Atlas). **(a)** Overall survival (OS) of all histological subgroups WHO grades from primary (left) (n=222) and recurrent (right) (n=58) gliomas; **(b)** OS of primary (left) (n=90) and recurrent (right) (n=4) WHO grade 2 gliomas; **(c)** OS of primary (left) (n=47) and recurrent (right) (n=27) WHO grade 3 gliomas; **(d)** OS of primary (left) (n=85) and recurrent (right) (n=23) WHO grade 4 gliomas.


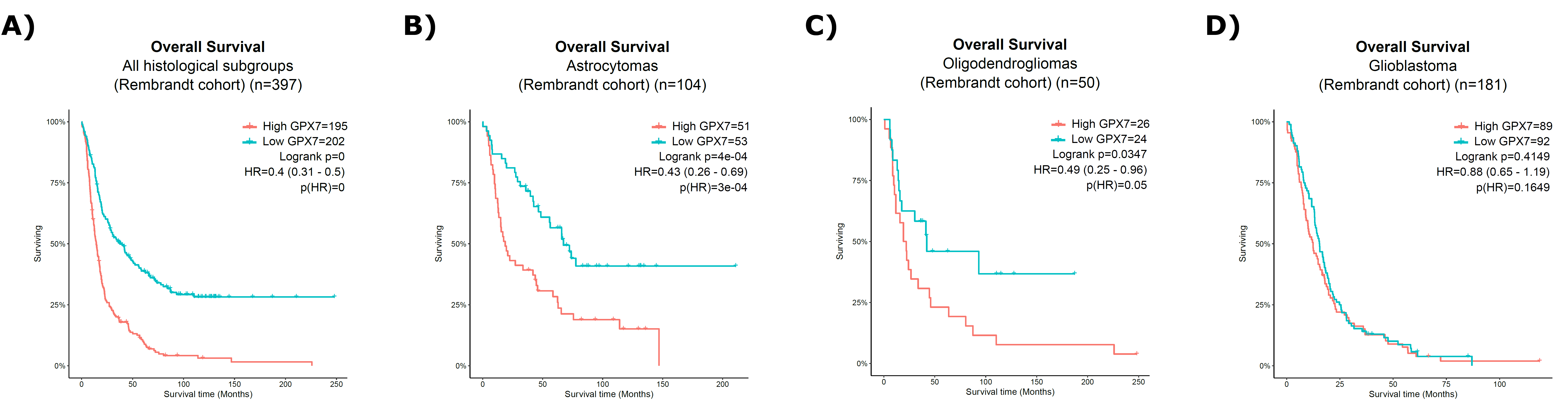
**Supplementary Figure S5:** Kaplan-Meier survival curves based on high (red) and low (green) expression of *GPX7* in gliomas from the Rembrandt cohort. **(a)** Overall survival (OS) of all histological subgroups (n=397) of gliomas; **(b)** OS of astrocytomas (n=104); **(c)** OS of oligodendrogliomas (n=50); **(d)** OS of glioblastoma (n=181).

**Supplementary Figure S6:** *GPX7* expression levels in a short-term cultured glioblastoma cell line (GLI56) treated with 5-aza-2'-deoxycytidine (5-aza-dC) (5μM) for 96h.


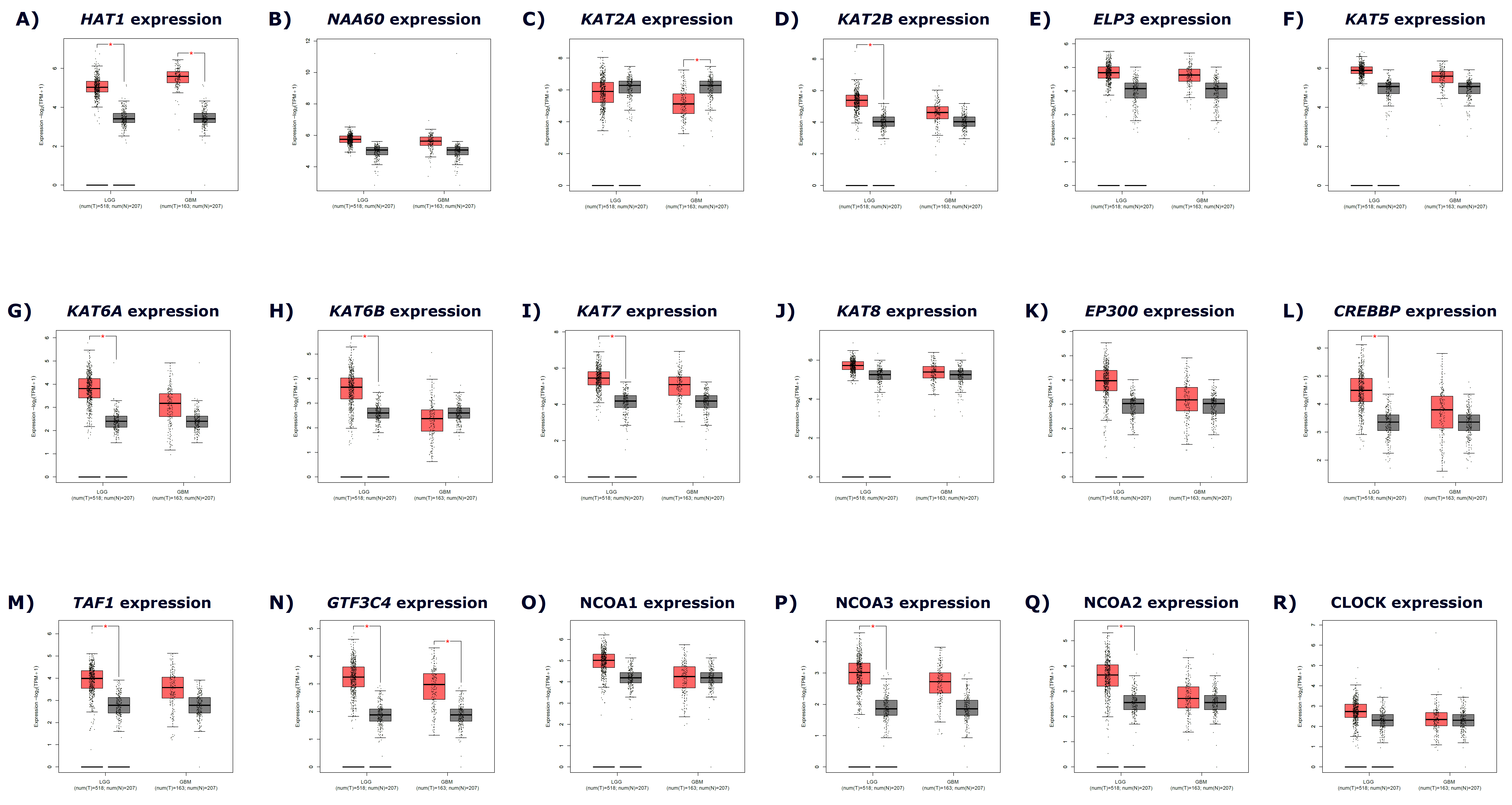
**Supplementary Figure S7:** Differential expression of histone acetyltransferases (HATs) between gliomas (LGG and GBM from TCGA) and normal human brain samples (from GTEx database). **(a)** *HAT1*, **(b)** *NAA60* **(c)** *KAT2A*, **(d)** *KAT2B,* **(e)** *ELP3,* **(f)** *KAT5*, **(g)** *KAT6A*, **(h)** *KAT6B*, **(i)** *KAT7*, **(j)** *KAT8*, **(k)** *EP300*, **(l)** *CREBBP*, **(m)** *TAF1*, **(n)** *GTF3C4*, **(o)** *NCOA1*, **(p)** *NCOA3*, **(q)** *NCOA2* and **(r)** *CLOCK*.


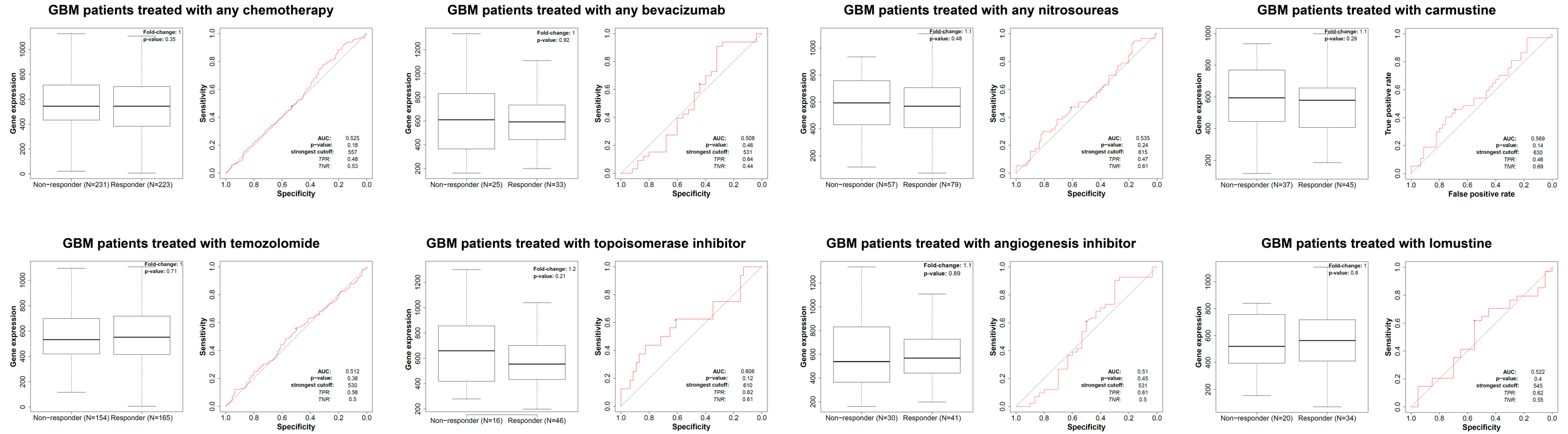
**Supplementary Figure S8:** GPX7 expression in GBM patients treated with multiple chemotherapeutic agents (Any chemotherapy, bevacizumab, nitrosoureas, carmustine, temozolomide, topoisomerase inhibitor, angiogenesis inhibitor and lomustine). Box plot depicting the expression of GPX7 (probe 213170_at) in GBM patients annotated with chemotherapy responses according to the Response Evaluation Criteria in Solid Tumours (RECIST) criteria. Graphs show normalized gene expression in irinotecan non-responders and responders patients. ROC plot of non-responders (N=10) and responders (N=23) of GBM patients treated with multiple chemotherapeutic agents based on overall survival (OS) at 16 months. Error bar ± SD. AUC: area under the curve. PPV, positive predictive value; TPR: true positive rate.


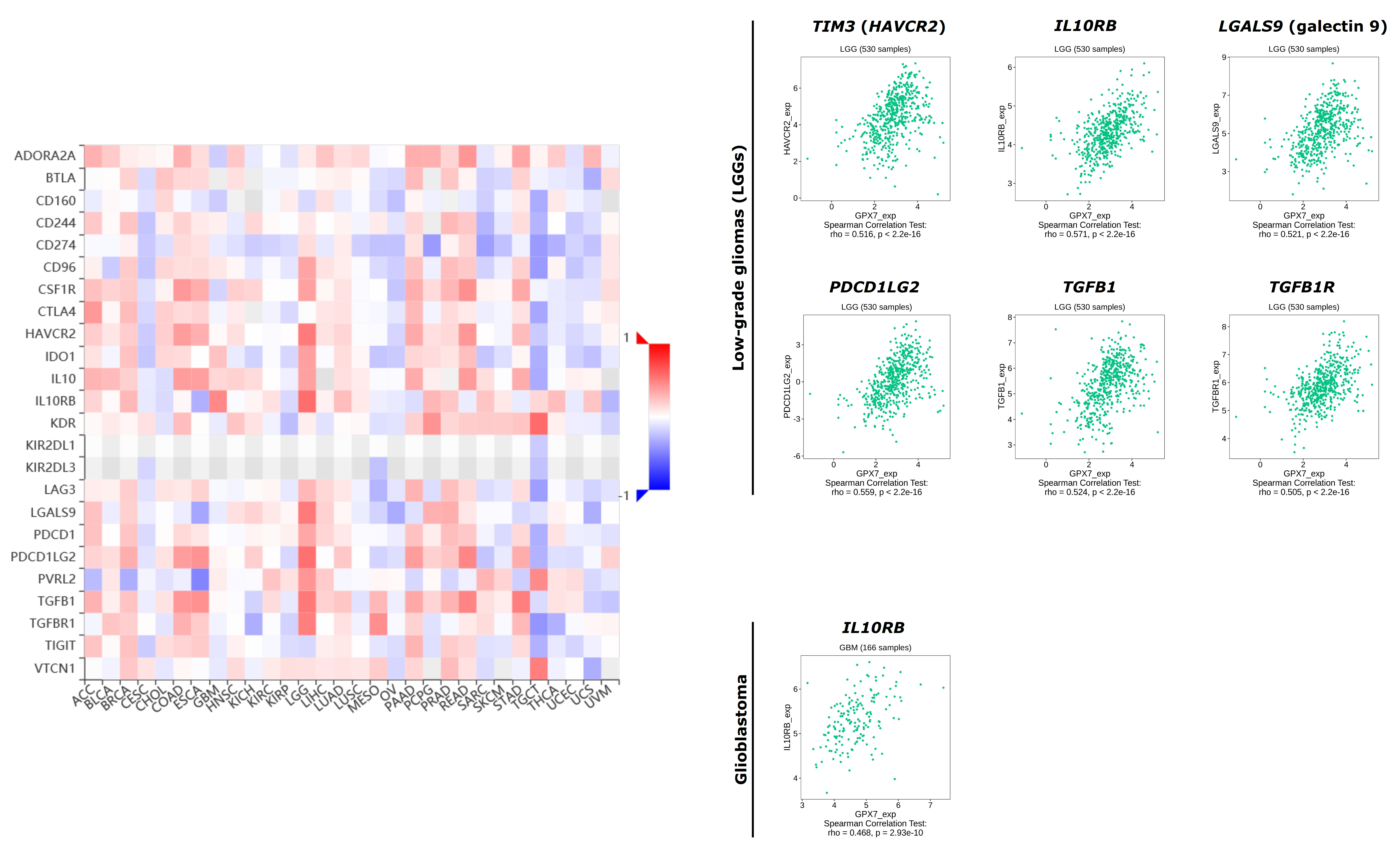
**Supplementary Figure S9:** Correlation between *GPX7* expression and immune checkpoint inhibitors. Left panel: Correlation heat map between *GPX7* expression and immune checkpoint inhibitors in pan-cancer. Upper panel: correlation between *GPX7* expression and (i) *TIM3* (*HAVCR2*); (ii) *IL10RB* (interleukin 10 receptor subunit beta); (iii) *LGALS9* (Galectin 9); (iv) *PDCD1LG2* (programmed cell death 1 ligand 2); (v) *TGFB1;* (vi) *TGFBR1* in low-grade gliomas (LGG). Lower panel: correlation between *GPX7* expression and *IL10RB* in glioblastoma (GBM).


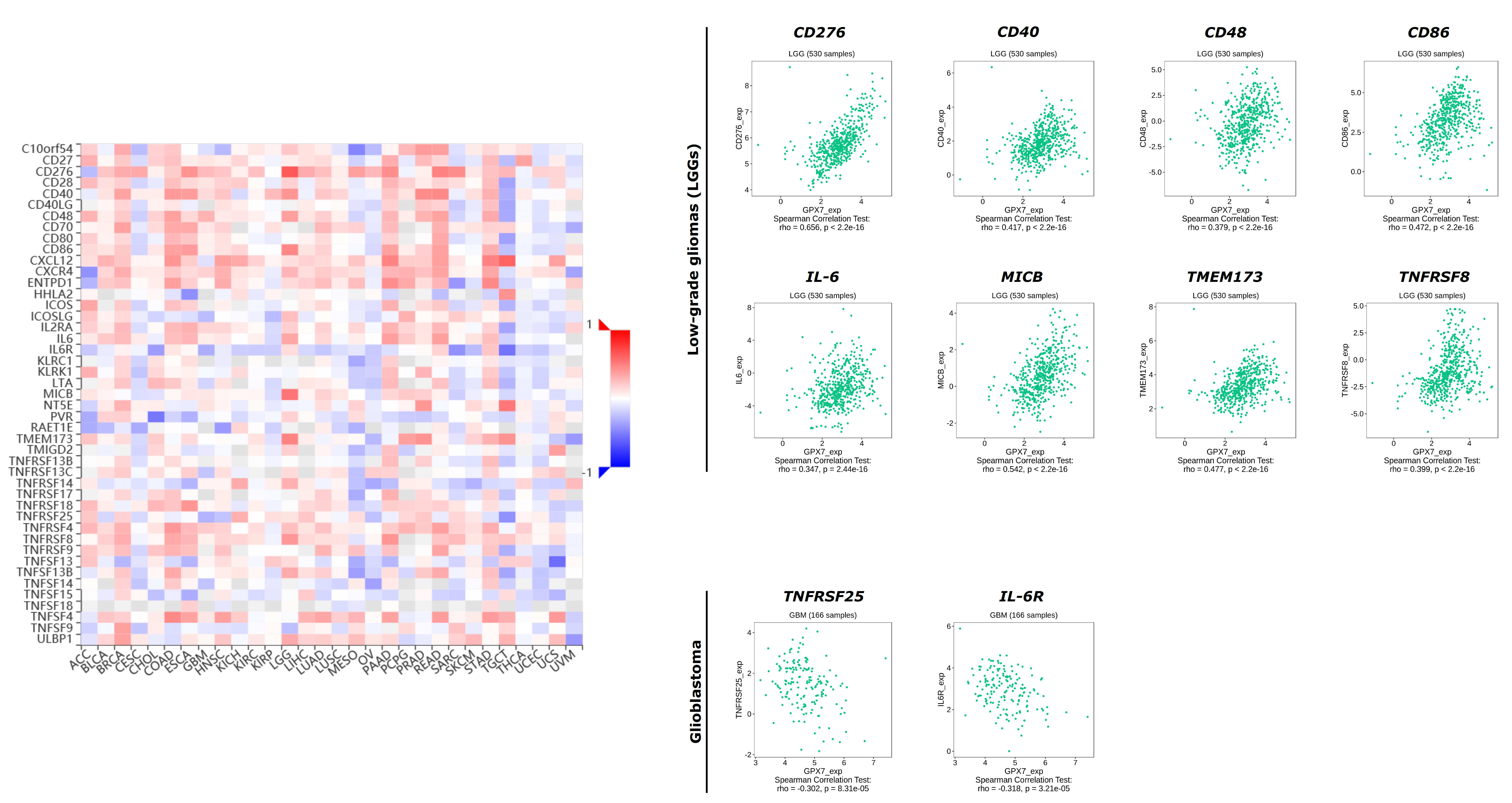
**Supplementary Figure S10:** Correlation between *GPX7* expression and immunostimulators. Left panel: Correlation heat map between *GPX7* expression and immunostimulators in pan-cancer. Upper panel: correlation between *GPX7* expression and (i) *CD276*; (ii) *CD40*; (iii) *CD48*; (iv) *CD86*; (v) *IL-6*; (vi) *MICB*; (vii) *TMEM173;* (viii) *TNFRSF8* in low-grade gliomas (LGG). Lower panel: correlation between *GPX7* expression and (i) *TNFRSF25;* (ii) *IL-6R* in glioblastoma (GBM).


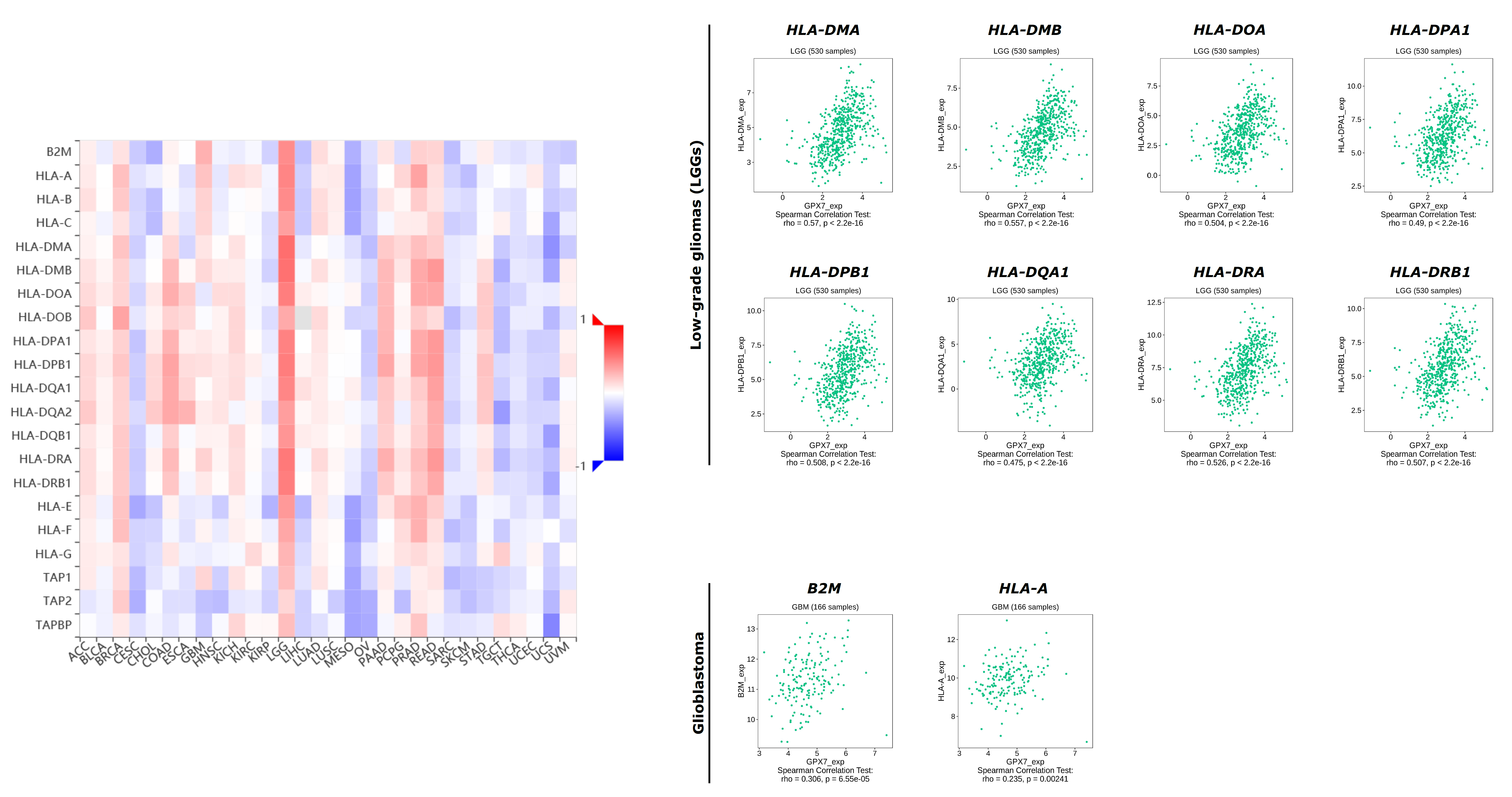


**Supplementary Figure S11:** Correlation between *GPX7* expression and MHC molecules. Left panel: Correlation heat map between *GPX7* expression and MHC molecules in pan-cancer. Upper panel: correlation between *GPX7* expression and (i) *HLA-DMA*; (ii) *HLA-DMB*; (iii) *HLA-DOA*; (iv) *HLA-DPA1*; (v) *HLA-DPB1*; (vi) *HLA-DQA1*; (vii) *HLA-DRA;* (viii) *HLA-DRB1* in low-grade gliomas (LGG). Lower panel: correlation between *GPX7* expression and (i) *B2M;* (ii) *HLA-A* in glioblastoma (GBM).

**Supplementary Tables**

**Supplementary Table S1:** Association of GPX7 expression and clinicopathological characteristics in glioma patients from TCGA database. Bold numbers indicate that they were statistically significant (p-value < 0.05).

| **Variables** | **Clinicopathological characteristics** | **Number** | **Mean±SD** | **P-value** |
| --- | --- | --- | --- | --- |
| **Histology** | Oligodendroglioma | 191 | 6.64±0.857 | **2.20E-16** |
|  | Oligoastrocytoma | 130 | 7.05±0.682 |  |
|  | Astrocytoma | 194 | 7.59±0.640 |  |
|  | Glioblastoma | 152 | 8.96±0.635 |  |
| **Histologic Grade** | II | 226 | 6.86±0.702 | **2.2E-16** |
|  | III | 244 | 7.34±0.887 |  |
|  | IV | 150 | 8.94±0.646 |  |
| **Gender** | Male | 579 | 6.99±0.811 | 0.7 |
|  | Female | 408 | 7.01±0.849 |  |
| **Additional pharmaceutical therapy** | YES | 156 | 7.36±0.754 | **5.40E-03** |
|  | NO | 105 | 7.06±0.782 |  |
| **Additional radiation therapy** | YES | 71 | 7.29±0.784 | 0.16 |
|  | NO | 190 | 7.20±0.790 |  |
| **Asthma history** | YES | 22 | 7.17±0.822 | 0.88 |
|  | NO | 353 | 7.11±0.851 |  |
| **Eczema history** | YES | 12 | 7.07±0.950 | 0.92 |
|  | NO | 353 | 7.12±0.846 |  |
| **Family history of cancer** | YES | 136 | 7.26±0833 | 0.17 |
|  | NO | 220 | 7.06±0.816 |  |
| **Family history of primary brain tumor** | YES | 14 | 7.40±0.753 | 0.42 |
|  | NO | 346 | 7.12±0.840 |  |
| **First presenting symptom** | Headaches | 110 | 7.20±0.806 | 0.3205 |
|  | Mental Status Changes | 42 | 7.09±0.875 |  |
|  | Motor/Movement Changes | 38 | 7.39±1.01 |  |
|  | Seizures | 262 | 7.09±0.855 |  |
|  | Sensory Changes | 19 | 7.18±0.695 |  |
|  | Visual Changes | 13 | 7.44±0.736 |  |
| **Followup treatment success** | Complete Remission/Response | 89 | 6.98±0.748 | **6.60E-06** |
|  | Partial Remission/Response | 17 | 7.04±0.970 |  |
|  | Progressive Disease | 113 | 7.46±0.794 |  |
|  | Stable Disease | 126 | 7.13±0.808 |  |
| **Food allergy history** | YES | 22 | 7.39±1.01 | 0.15 |
|  | NO | 298 | 7.09±0.848 |  |
| **Hay fever history** | YES | 38 | 6.78±0.957 | 0.15 |
|  | NO | 316 | 7.14±0.818 |  |
| **Headache history** | YES | 177 | 7.18±0.854 | 0.19 |
|  | NO | 302 | 7.11±0.834 |  |
| **History ionizing RT to head** | YES | 4 | 7.19±0.933 | 0.44 |
|  | NO | 505 | 7.13±0.849 |  |
| **Laterality** | Right | 260 | 7.12±0.859 | 0.93 |
|  | Left | 257 | 7.12±0.818 |  |
|  | Midline | 7 | 7.18±1.13 |  |
| **Lost follow up** | YES | 60 | 7.17±0.914 | 0.18 |
|  | NO | 316 | 7.06±0.820 |  |
| **Mental status changes** | YES | 120 | 7.14±0.842 | 0.85 |
|  | NO | 353 | 7.12±0.827 |  |
| **Mold or dust allergy history** | YES | 18 | 7.07±0.818 | 0.73 |
|  | NO | 319 | 7.10±0.856 |  |
| **Motor movement changes** | YES | 113 | 7.27±0.827 | 0.07 |
|  | NO | 357 | 7.10±0.837 |  |
| **New tumor event after initial treatment** | YES | 159 | 7.36±0.841 | **2.90E-06** |
|  | NO | 301 | 6.96±0.841 |  |
| **Postoperative rx tx** | YES | 207 | 7.18±0.883 | **3.80E-02** |
|  | NO | 255 | 6.97±0.741 |  |
| **Preoperative antiseizure meds** | YES | 273 | 7.11±0.827 | 0.49 |
|  | NO | 114 | 7.18±0.758 |  |
| **Preoperative corticosteroids** | YES | 155 | 7.16±0.780 | 0.69 |
|  | NO | 222 | 7.08±0.801 |  |
| **Primary therapy outcome success** | Complete Remission/Response | 135 | 6.99±0.767 | **3.80E-05** |
|  | Partial Remission/Response | 65 | 6.95±0.789 |  |
|  | Progressive Disease | 116 | 7.40±0.832 |  |
|  | Stable Disease | 138 | 6.99±0.836 |  |
| **Radiation therapy** | YES | 429 | 7.27±0.776 | **1.30E-09** |
|  | NO | 206 | 6.80±0.823 |  |
| **Seizure history** | YES | 311 | 7.27±0.776 | 0.19 |
|  | NO | 183 | 6.80±0.823 |  |
| **Sensory changes** | YES | 75 | 7.40±0.740 | **2.70E-03** |
|  | NO | 392 | 7.08±0.841 |  |
| **Supratentorial localization** | Cerebral Cortex | 142 | 6.98±0.857 | 0.52 |
|  | Deep Gray (e.g.basal ganglia, thalamus) | 3 | 7.73±0.965 |  |
|  | White Matter | 124 | 7.05±0.757 |  |
| **Tumor location** | Posterior Fossa, Brain Stem | 2 | 8.42±0.008 | 0.10 |
|  | Posterior Fossa, Cerebellum | 2 | 7.65±0.009 |  |
|  | Supratentorial, Frontal Lobe | 314 | 7.02±0.852 |  |
|  | Supratentorial, Occipital Lobe | 8 | 7.38±1.07 |  |
|  | Supratentorial, Parietal Lobe | 49 | 7.16±0.827 |  |
|  | Supratentorial, Temporal Lobe | 151 | 7.26±0.811 |  |
| **Visual changes** | YES | 67 | 7.38±0.718 | **1.00E-02** |
|  | NO | 405 | 7.10±0.846 |  |
| **Race** | American indian or alaska native | 1 | 7.65±0 | 0.53 |
|  | Asian | 8 | 7.16±1.11 |  |
|  | Black or african american | 22 | 6.87±0.755 |  |
|  | White | 496 | 6.72±1.48 |  |

**Supplementary Table S2:** Association of GPX7 expression and clinicopathological characteristics in glioma patients from CGGA database. Bold numbers indicate that they were statistically significant (p-value < 0.05).

| **Variables** | **Clinicopathological characteristics** | **Number** | **Mean±SD** | **P-value** |
| --- | --- | --- | --- | --- |
| **Radiation therapy** | YES | 517 | 3.06±1.17 | **3.50E-02** |
|  | NO | 100 | 2.80±1.17 |  |
| **Chemotherapy** | YES | 635 | 3.12±1.20 | **4.40E-03** |
|  | NO | 275 | 2.76±1.11 |  |
| **Progression Status** | Primary | 422 | 8.33±8.12 | **8.40E-03** |
|  | Recurrent | 270 | 11.3±9.23 |  |
| **Histology** | Anaplastic astrocytoma | 82 | 9.13±5.65 | **9.40E-38** |
|  | Anaplastic Oligoastrocytoma | 16 | 2.10±1.56 |  |
|  | Anaplastic Oligodendroglioma | 46 | 5.14±3.55 |  |
|  | Astrocytoma | 85 | 6.79±9.76 |  |
|  | GBM | 140 | 12.4±9.05 |  |
|  | Oligoastrocytoma | 8 | 1.97±1.48 |  |
|  | Oligodendroglioma | 45 | 3.57±1.86 |  |
|  | Recurrent Anaplastic Astrocytoma | 70 | 13.2±10.1 |  |
|  | Recurrent Anaplastic Oligoastrocytoma | 5 | 2.70±3.71 |  |
|  | Recurrent Anaplastic Oligodendroglioma | 36 | 6.68±5.14 |  |
|  | Recurrent Astrocytoma | 34 | 7.71±4.27 |  |
|  | Recurrent GBM | 109 | 13.8±10.2 |  |
|  | Recurrent Oligoastrocytoma | 1 | 5.59±- |  |
|  | Recurrent Oligodendroglioma | 15 | 6.45±3.60 |  |
| **Gender** | Male | 393 | 9.26±8.81 | 6.80E-01 |
|  | Female | 289 | 9.84±8.61 |  |

**Supplementary Table S3:** Overall survival analysis of all CpG sites from *GPX7* by Kaplan-Meier test using MethSurv. HR, hazard ratio; LR, log‑likelihood ratio. Bold numbers indicate that they were statistically significant (p-value < 0.05).

|  | **Overall survival (OS)** | | | | | | | | |
| --- | --- | --- | --- | --- | --- | --- | --- | --- | --- |
| **Tumor** | **Relation to island** | **Genomic Region** | **CpG site** | **Hazard ratio (HR)** | **CI** | **LR test P value** | **Mean** | **Median** | **Range** |
| **LGG** | Open Sea | 3'UTR | cg00998379 | 0.764 | (0.536;1.089) | 0.14 | 0.95 | 0.95 | (0.676;0.974) |
|  | Island | Body | cg02453146 | 0.386 | (0.267;0.556) | **1.70E-07** | 0.13 | 0.08 | (0.036;0.748) |
|  |  |  | cg23272399 | 0.367 | (0.252;0.532) | **4.70E-08** | 0.1 | 0.03 | (0.012;0.805) |
|  |  | TSS200 | cg11953272 | 0.679 | (0.477;0.967) | **0.032** | 0.04 | 0.03 | (0.016;0.625) |
|  |  |  | cg22129364 | 0.71 | (0.497;1.014) | 0.058 | 0.1 | 0.06 | (0.021;0.816) |
|  |  | 1stExon; 5′UTR | cg16557944 | 0.729 | (0.512;1.038) | 0.078 | 0.06 | 0.04 | (0.019;0.806) |
|  |  |  | cg20950465 | 0.88 | (0.617;1.255) | 0.48 | 0.05 | 0.03 | (0.01;0.704) |
|  | S-Shelf | Body | cg03330022 | 0.685 | (0.479;0.98) | **0.037** | 0.86 | 0.89 | (0.314;0.962) |
|  | S-Shore | Body | cg09161043 | 0.451 | (0.305;0.667) | **3.00E-13** | 0.73 | 0.79 | (0.15;0.942) |
|  | N-Shore | TSS1500 | cg18087326 | 0.411 | (0.285;0.592) | **1.30E-06** | 0.78 | 0.81 | (0.262;0.94) |
|  |  |  | cg26251270 | 0.401 | (0.278;0.579) | **5.90E-07** | 0.06 | 0.03 | (0.02;0.81) |
|  |  |  |  |  |  |  |  |  |  |
| **GBM** | Open Sea | 3'UTR | cg00998379 | 0.853 | (0.567;1.282) | 0.44 | 0.93 | 0.95 | (0.645;0.97) |
|  | Island | Body | cg02453146 | 0.621 | (0.404;0.956) | **0.029** | 0.08 | 0.07 | (0.037;0.5) |
|  |  |  | cg23272399 | 0.865 | (0.575;1.303) | 0.49 | 0.04 | 0.03 | (0.013;0.478) |
|  |  | TSS200 | cg11953272 | 1.099 | (0.731;1.653) | 0.65 | 0.03 | 0.03 | (0.017;0.217) |
|  |  |  | cg22129364 | 1.041 | (0.692;1.568) | 0.85 | 0.06 | 0.05 | (0.022;0.453) |
|  |  | 1stExon; 5′UTR | cg16557944 | 0.707 | (0.469;1.065) | 0.097 | 0.04 | 0.04 | (0.017;0.501) |
|  |  |  | cg20950465 | 0.726 | (0.477;1.104) | 0.13 | 0.04 | 0.03 | (0.01;0.609) |
|  | S-Shore | Body | cg09161043 | 0.907 | (0.603;1.365) | 0.64 | 0.52 | 0.53 | (0.068;0.897) |
|  | N-Shore | TSS1500 | cg18087326 | 0.748 | (0.494;1.134) | 0.17 | 0.67 | 0.7 | (0.127;0.899) |
|  |  |  | cg26251270 | 0.876 | (0.582;1.318) | 0.53 | 0.03 | 0.03 | (0.017;0.421) |

**Supplementary Table S4:** Spearman’s correlation between the expression of *GPX7* and histone acetyltransferases (HATs) in gliomas from the TCGA database. Bold numbers indicate that they were statistically significant (p-value < 0.05).

|  |  |  |  |  | **LGG Tumors** | | **Glioblastomas** | |
| --- | --- | --- | --- | --- | --- | --- | --- | --- |
| **Type** | **Cell Location** | **Family** | **Gene Name (Official Symbol)** | **Other names** | **Correlation (Spearman's rho)** | **p-value** | **Correlation (Spearman's rho )** | **p-value** |
| **Type B** | **Cytoplasm** | **Cytoplasmatic** | HAT1 | KAT1 | 0.71 | **1.00E-51** | 0.31 | **5.50E-05** |
|  |  |  | NAA60 | HAT4; NatF; NAT15; hNaa60 | 0.32 | **1.40E-13** | -0.087 | 0.27 |
| **Type A** | **Nucleus** | **GNAT** | KAT2A | GCN5; GCN5L2; PCAF-b; hGCN5 | -0.11 | **0.0091** | 0.0072 | 0.93 |
|  |  |  | KAT2B | CAF; P/CAF; PCAF | -0.038 | 0.39 | -0.15 | 0.05 |
|  |  |  | ELP3 | KAT9 | -0.026 | 0.55 | 0.12 | 0.14 |
|  |  | **MYST** | KAT5 | ESA1; HTATIP; HTATIP1; NEDFASB; PLIP; TIP; TIP60; ZC2HC5; cPLA2 | -0.021 | 0.63 | 0.051 | 0.51 |
|  |  |  | KAT6A | ARTHS; MOZ; MRD32; MYST-3; MYST3; RUNXBP2; ZC2HC6A; ZNF220 | 0.13 | **0.0023** | -0.093 | 0.24 |
|  |  |  | KAT6B | GTPTS; MORF; MOZ2; MYST4; ZC2HC6B; qkf; querkopf | -0.12 | **0.0077** | -0.1 | 0.19 |
|  |  |  | KAT7 | HBO1; HBOA; MYST2; ZC2HC7 | 0.045 | 0.31 | 0.048 | 0.55 |
|  |  |  | KAT8 | LIGOWS; MOF; MYST1; ZC2HC8; hMOF | 0.096 | **0.029** | -0.046 | 0.56 |
|  |  | **p300/CBP** | EP300 | p300; KAT3B; MKHK2; RSTS2 | 0.047 | 0.28 | -0.14 | 0.081 |
|  |  |  | CREBBP | CBP; RSTS; KAT3A; MKHK1; RSTS1 | 0.066 | 0.13 | -0.18 | **0.024** |
|  |  | **Transcription co-activators** | TAF1 | OF; XDP; BA2R; CCG1; CCGS; DYT3; KAT4; P250; NSCL2; TAF2A; MRXS33; N-TAF1; TAFII250; DYT3/TAF1; TAFII-250; TAF(II)250 | 0.2 | **2.60E-06** | -0.056 | 0.48 |
|  |  |  | GTF3C4 | KAT12; TFIII90; TFIIIC90; TFIIIC290; TF3C-delta; TFIIICDELTA | 0.18 | **2.50E-05** | -0.027 | 0.73 |
|  |  | **Steroid receptor co-activators** | NCOA1 | SRC1; KAT13A; RIP160; F-SRC-1; bHLHe42; bHLHe74 | 0.0099 | 0.82 | -0.23 | **0.0033** |
|  |  |  | NCOA3 | ACTR; AIB1; RAC3; SRC3; pCIP; AIB-1; CTG26; SRC-3; CAGH16; KAT13B; TNRC14; TNRC16; TRAM-1; bHLHe42 | 0.29 | **1.10E-11** | -0.0049 | 0.95 |
|  |  |  | NCOA2 | SRC2; TIF2; GRIP1; KAT13C; NCoA-2; bHLHe75 | -0.074 | 0.092 | -0.2 | **0.0091** |
|  |  |  | CLOCK | KAT13D; bHLHe8 | 0.13 | **0.0034** | -0.1 | 0.21 |

**Supplementary Table S5:** Correlation between the expression of *GPX7* and miRNAs in LGG patients from TCGA database using the Multi-Scale Association Explorer (MSAE) tool on the Cancer Regulome.

| **miRNAs** | **Chromosome** | **Start** | **End** | **Correlation** | **Adjusted logged pvalue (-LOG10(p))** | **Logged pvalue (-LOG10(p))** |
| --- | --- | --- | --- | --- | --- | --- |
| hsa-mir-24-2-5p | 19 | 13947101 | 13947173 | 0.501 | 10.9 | 17.9 |
| hsa-mir-1262 | 1 | 68649201 | 68649293 | 0.479 | 9.2 | 16.2 |
| hsa-mir-21-3p | 17 | 57918627 | 57918698 | 0.468 | 8.4 | 15.4 |
| hsa-mir-92b-5p | 1 | 155164968 | 155165063 | 0.467 | 8.3 | 15.3 |
| hsa-mir-21-5p | 17 | 57918627 | 57918698 | 0.448 | 7 | 14 |
| hsa-mir-196b-5p | 7 | 27209099 | 27209182 | 0.437 | 6.3 | 13.3 |
| hsa-mir-505-3p | x | 139006307 | 139006390 | 0.417 | 5.1 | 12.1 |
| hsa-mir-219-1-3p | 6 | 33175612 | 33175721 | 0.414 | 4.9 | 11.9 |
| hsa-mir-2114-3p | x | 149396239 | 149396318 | 0.407 | 4.5 | 11.5 |
| hsa-mir-27a-5p | 19 | 13947254 | 13947331 | 0.396 | 3.8 | 10.9 |
| hsa-mir-10b-5p | 2 | 177015031 | 177015140 | 0.395 | 3.8 | 10.8 |
| hsa-mir-196a-5p | 12 | 54385522 | 54385631 | 0.385 | 3.2 | 10.3 |
| hsa-mir-10b-3p | 2 | 177015031 | 177015140 | 0.374 | 2.6 | 9.7 |
| hsa-mir-296-3p | 20 | 57392670 | 57392749 | 0.371 | 2.5 | 9.5 |
| hsa-mir-155-5p | 21 | 26946292 | 26946356 | 0.357 | 1.8 | 8.8 |
| hsa-mir-320c | 18 | 19263471 | 19263558 | 0.355 | 1.7 | 8.8 |
| hsa-mir-3677-3p | 16 | 2320714 | 2320773 | 0.354 | 1.7 | 8.7 |
| hsa-mir-125b-1-3p | 11 | 121970465 | 121970552 | 0.351 | 1.5 | 8.6 |
| hsa-mir-23a-3p | 19 | 13947401 | 13947473 | 0.349 | 1.4 | 8.5 |
| hsa-mir-516a-5p | 19 | 54259995 | 54260084 | -0.349 | 1.4 | 8.4 |
| hsa-mir-330-3p | 19 | 46142252 | 46142345 | -0.351 | 1.5 | 8.6 |
| hsa-mir-1179 | 15 | 89151338 | 89151428 | -0.352 | 1.6 | 8.6 |
| hsa-mir-138-2-3p | 16 | 56892430 | 56892513 | -0.354 | 1.7 | 8.7 |
| hsa-mir-129-1-3p | 7 | 127847925 | 127847996 | -0.354 | 1.6 | 8.7 |
| hsa-mir-885-5p | 3 | 10436173 | 10436246 | -0.355 | 1.7 | 8.7 |
| hsa-mir-504 | x | 137749872 | 137749954 | -0.357 | 1.8 | 8.8 |
| hsa-mir-490-5p | 7 | 136587914 | 136588041 | -0.357 | 1.8 | 8.8 |
| hsa-mir-433 | 14 | 101348223 | 101348315 | -0.358 | 1.9 | 8.9 |
| hsa-mir-29c-3p | 1 | 207975197 | 207975284 | -0.36 | 1.9 | 9 |
| hsa-mir-133b | 6 | 52013721 | 52013839 | -0.361 | 2 | 9 |
| hsa-mir-323a-5p | 14 | 101492069 | 101492154 | -0.361 | 2 | 9 |
| hsa-mir-338-3p | 17 | 79099683 | 79099749 | -0.364 | 2.2 | 9.2 |
| hsa-mir-873-5p | 9 | 28888877 | 28888953 | -0.368 | 2.3 | 9.4 |
| hsa-mir-519a-5p | 19 | 54255651 | 54255735 | -0.371 | 2.5 | 9.5 |
| hsa-mir-218-5p | 4 | 20529898 | 20530007 | -0.373 | 2.6 | 9.7 |
| hsa-mir-1258 | 2 | 180725563 | 180725635 | -0.379 | 2.9 | 10 |
| hsa-mir-885-3p | 3 | 10436173 | 10436246 | -0.385 | 3.2 | 10.3 |
| hsa-mir-138-1-3p | 3 | 44155704 | 44155802 | -0.386 | 3.3 | 10.3 |
| hsa-mir-7-2-3p | 15 | 89155056 | 89155165 | -0.39 | 3.5 | 10.5 |
| hsa-mir-129-5p | 11 | 43602944 | 43603033 | -0.397 | 3.9 | 11 |
| hsa-mir-219-2-3p | 9 | 131154897 | 131154993 | -0.398 | 4 | 11 |
| hsa-mir-491-5p | 9 | 20716104 | 20716187 | -0.402 | 4.2 | 11.2 |
| hsa-mir-1 | 18 | 19408965 | 19409049 | -0.402 | 4.2 | 11.2 |
| hsa-mir-124-3p | 20 | 61809852 | 61809938 | -0.408 | 4.6 | 11.6 |
| hsa-mir-203a | 14 | 104583742 | 104583851 | -0.411 | 4.7 | 11.8 |
| hsa-mir-346 | 10 | 88024451 | 88024545 | -0.415 | 4.9 | 12 |
| hsa-mir-490-3p | 7 | 136587914 | 136588041 | -0.415 | 4.9 | 12 |
| hsa-mir-1224-3p | 3 | 183959193 | 183959277 | -0.417 | 5 | 12.1 |
| hsa-mir-767-3p | x | 151561893 | 151562001 | -0.422 | 5.4 | 12.4 |
| hsa-mir-1249 | 22 | 45596835 | 45596900 | -0.431 | 6 | 13 |
| hsa-mir-874 | 5 | 136983261 | 136983338 | -0.434 | 6.1 | 13.2 |
| hsa-mir-138-5p | 16 | 56892430 | 56892513 | -0.434 | 6.1 | 13.2 |
| hsa-mir-133a | 18 | 19405659 | 19405746 | -0.448 | 7 | 14 |
| hsa-mir-105-5p | x | 151560691 | 151560771 | -0.449 | 7.1 | 14.1 |
| hsa-mir-1224-5p | 3 | 183959193 | 183959277 | -0.451 | 7.2 | 14.3 |
| hsa-mir-137 | 1 | 98511626 | 98511727 | -0.476 | 9 | 16 |
| hsa-mir-767-5p | x | 151561893 | 151562001 | -0.49 | 10 | 17 |
| hsa-mir-3943 | 7 | 43190494 | 43190593 | -0.506 | 11.3 | 18.3 |
| hsa-mir-124-5p | 20 | 61809852 | 61809938 | -0.52 | 12.4 | 19.4 |
| hsa-mir-139-5p | 11 | 72326107 | 72326174 | -0.521 | 12.5 | 19.6 |
| hsa-mir-139-3p | 11 | 72326107 | 72326174 | -0.523 | 12.7 | 19.7 |
| hsa-mir-128 | 2 | 136422967 | 136423048 | -0.547 | 14.8 | 21.9 |

**Supplementary Table S6:** Correlation between the expression of *GPX7* and miRNAs in GBM patients from TCGA database using the Multi-Scale Association Explorer (MSAE) tool on the Cancer Regulome.

| **miRNAs** | **Chromosomal location** | **Start** | **End** | **Correlation** | **Adjusted logged pvalue (-LOG10(p))** | **Logged pvalue (-LOG10(p))** |
| --- | --- | --- | --- | --- | --- | --- |
| hsa-miR-768-3p | 16 | 70349796 | 70349899 | -0.29 | 3.5 | 10.2 |
| hsa-miR-492 | 12 | 93752305 | 93752420 | 0.243 | 0.6 | 7.3 |
| hsa-miR-29c | 1 | 206041820 | 206041907 | -0.241 | 0.5 | 7.2 |
| hsa-let-7e | 19 | 56887851 | 56887929 | -0.238 | 0.3 | 7 |
| hsa-miR-139 | 11 | 72003755 | 72003822 | -0.237 | 0.3 | 6.9 |
| hsa-miR-629 | 15 | 68158765 | 68158861 | 0.235 | 0.2 | 6.9 |
| hsa-miR-503 | X | 133508024 | 133508094 | 0.235 | 0.2 | 6.8 |
| hsa-miR-29b |  |  |  | -0.234 | 0.1 | 6.8 |
| hsa-miR-199a |  |  |  | 0.233 | 0.1 | 6.8 |
| hsa-miR-30e-5p | 1 | 40992614 | 40992705 | -0.226 | 0 | 6.4 |
| hsa-miR-199b | 9 | 130046821 | 130046930 | 0.219 | 0 | 6 |

**Supplementary Table S7:** Spearman’s correlation between the expression of *GPX7* and immune infiltrates' abundances estimated by multiple immune deconvolution methods (TIMER, CIBERSORT, quanTIseq, xCell, MCP-counter and EPIC algorithms).

| **Cancer** | **Infiltrates** | **Spearman's Rho** | **p** | **Adjusted P-values** |
| --- | --- | --- | --- | --- |
| LGG (n=516) | B cell memory_CIBERSORT | 0.113643606 | 0.012912703 | 0.049169254 |
| LGG (n=516) | B cell memory_CIBERSORT-ABS | 0.127537498 | 0.0052304 | 0.023491917 |
| LGG (n=516) | B cell memory_XCELL | 0.067662533 | 0.139638036 | 0.297404974 |
| LGG (n=516) | B cell naive_CIBERSORT | -0.162861424 | 0.000349895 | 0.002415491 |
| LGG (n=516) | B cell naive_CIBERSORT-ABS | -0.051620337 | 0.260003641 | 0.456652596 |
| LGG (n=516) | B cell naive_XCELL | 0.007606506 | 0.868259541 | 0.93101669 |
| LGG (n=516) | B cell plasma_CIBERSORT | -0.45170704 | 2.07E-25 | 2.50E-23 |
| LGG (n=516) | B cell plasma_CIBERSORT-ABS | -0.334075535 | 6.31E-14 | 2.51E-12 |
| LGG (n=516) | B cell plasma_XCELL | -0.277259045 | 6.95E-10 | 1.48E-08 |
| LGG (n=516) | B cell_EPIC | -0.373955301 | 2.60E-17 | 1.50E-15 |
| LGG (n=516) | B cell_MCPCOUNTER | -0.098795718 | 0.030801935 | 0.096232394 |
| LGG (n=516) | B cell_QUANTISEQ | -0.12396023 | 0.006656733 | 0.028646822 |
| LGG (n=516) | B cell_TIMER | 0.189046202 | 3.18E-05 | 0.000287422 |
| LGG (n=516) | B cell_XCELL | 0.247445634 | 4.22E-08 | 6.64E-07 |
| LGG (n=516) | Cancer associated fibroblast_EPIC | 0.128488309 | 0.004900804 | 0.022164999 |
| LGG (n=516) | Cancer associated fibroblast_MCPCOUNTER | 0.305493263 | 8.77E-12 | 2.42E-10 |
| LGG (n=516) | Cancer associated fibroblast_TIDE | 0.319111769 | 8.92E-13 | 2.92E-11 |
| LGG (n=516) | Cancer associated fibroblast_XCELL | 0.236376973 | 1.71E-07 | 2.43E-06 |
| LGG (n=516) | Class-switched memory B cell_XCELL | 0.047703098 | 0.297966124 | 0.499374203 |
| LGG (n=516) | Common lymphoid progenitor_XCELL | 0.386701846 | 1.69E-18 | 1.11E-16 |
| LGG (n=516) | Common myeloid progenitor_XCELL | -0.008967509 | 0.844963069 | 0.917531665 |
| LGG (n=516) | Endothelial cell_EPIC | 0.026344752 | 0.565580413 | 0.730847417 |
| LGG (n=516) | Endothelial cell_MCPCOUNTER | -0.025513977 | 0.577904921 | 0.740916196 |
| LGG (n=516) | Endothelial cell_XCELL | 0.099279794 | 0.02998744 | 0.094396501 |
| LGG (n=516) | Eosinophil_CIBERSORT | 0.001207352 | 0.978996127 | 0.988633302 |
| LGG (n=516) | Eosinophil_CIBERSORT-ABS | 0.01527163 | 0.739110041 | 0.855274053 |
| LGG (n=516) | Eosinophil_XCELL | 0.067440403 | 0.140945312 | 0.299214608 |
| LGG (n=516) | Granulocyte-monocyte progenitor_XCELL | 0.031520672 | 0.491761634 | 0.67288281 |
| LGG (n=516) | Hematopoietic stem cell_XCELL | -0.03948573 | 0.389039498 | 0.58842888 |
| LGG (n=516) | Macrophage M0_CIBERSORT | -0.056519333 | 0.217409815 | 0.404177385 |
| LGG (n=516) | Macrophage M0_CIBERSORT-ABS | -0.055343445 | 0.227146057 | 0.415281393 |
| LGG (n=516) | Macrophage M1_CIBERSORT | 0.148303819 | 0.00114633 | 0.006752578 |
| LGG (n=516) | Macrophage M1_CIBERSORT-ABS | 0.208275022 | 4.39E-06 | 4.90E-05 |
| LGG (n=516) | Macrophage M1_QUANTISEQ | 0.335334565 | 5.02E-14 | 2.03E-12 |
| LGG (n=516) | Macrophage M1_XCELL | 0.525970148 | 2.32E-35 | 5.05E-33 |
| LGG (n=516) | Macrophage M2_CIBERSORT | 0.354161054 | 1.43E-15 | 6.95E-14 |
| LGG (n=516) | Macrophage M2_CIBERSORT-ABS | 0.5050259 | 2.65E-32 | 4.66E-30 |
| LGG (n=516) | Macrophage M2_QUANTISEQ | 0.490141678 | 2.96E-30 | 4.37E-28 |
| LGG (n=516) | Macrophage M2_TIDE | -0.406202822 | 2.05E-20 | 1.64E-18 |
| LGG (n=516) | Macrophage M2_XCELL | 0.084828685 | 0.063866172 | 0.168384486 |
| LGG (n=516) | Macrophage_EPIC | 0.459529515 | 2.40E-26 | 3.05E-24 |
| LGG (n=516) | Macrophage_TIMER | 0.147333045 | 0.001236233 | 0.007167519 |
| LGG (n=516) | Macrophage_XCELL | 0.450721328 | 2.71E-25 | 3.10E-23 |
| LGG (n=516) | Macrophage/Monocyte_MCPCOUNTER | 0.164268494 | 0.000310281 | 0.002194983 |
| LGG (n=516) | Mast cell activated_CIBERSORT | 0.120473652 | 0.008372814 | 0.034455507 |
| LGG (n=516) | Mast cell activated_CIBERSORT-ABS | 0.15226442 | 0.000838488 | 0.005130693 |
| LGG (n=516) | Mast cell resting_CIBERSORT | -0.109506707 | 0.016615529 | 0.059740203 |
| LGG (n=516) | Mast cell resting_CIBERSORT-ABS | -0.049673855 | 0.278428779 | 0.4767559 |
| LGG (n=516) | Mast cell_XCELL | 0.319144968 | 8.87E-13 | 2.92E-11 |
| LGG (n=516) | MDSC_TIDE | 0.049867452 | 0.276557517 | 0.474973267 |
| LGG (n=516) | Monocyte_CIBERSORT | 0.002122923 | 0.963077156 | 0.981956815 |
| LGG (n=516) | Monocyte_CIBERSORT-ABS | 0.234241598 | 2.22E-07 | 3.07E-06 |
| LGG (n=516) | Monocyte_MCPCOUNTER | 0.164268494 | 0.000310281 | 0.002194983 |
| LGG (n=516) | Monocyte_QUANTISEQ | -0.169110136 | 0.000203689 | 0.001503688 |
| LGG (n=516) | Monocyte_XCELL | 0.381992531 | 4.70E-18 | 2.87E-16 |
| LGG (n=516) | Myeloid dendritic cell activated_CIBERSORT | 0.106756469 | 0.019564146 | 0.068146953 |
| LGG (n=516) | Myeloid dendritic cell activated_CIBERSORT-ABS | 0.11076043 | 0.015405776 | 0.056364699 |
| LGG (n=516) | Myeloid dendritic cell activated_XCELL | 0.534661364 | 1.08E-36 | 2.60E-34 |
| LGG (n=516) | Myeloid dendritic cell resting_CIBERSORT | 0.033220413 | 0.468695439 | 0.657639185 |
| LGG (n=516) | Myeloid dendritic cell resting_CIBERSORT-ABS | 0.038722238 | 0.398282418 | 0.597541225 |
| LGG (n=516) | Myeloid dendritic cell_MCPCOUNTER | 0.382179826 | 4.52E-18 | 2.79E-16 |
| LGG (n=516) | Myeloid dendritic cell_QUANTISEQ | 0.072082485 | 0.115516051 | 0.25841494 |
| LGG (n=516) | Myeloid dendritic cell_TIMER | 0.572286845 | 6.20E-43 | 2.58E-40 |
| LGG (n=516) | Myeloid dendritic cell_XCELL | -0.02637332 | 0.56515885 | 0.730508912 |
| LGG (n=516) | Neutrophil_CIBERSORT | 0.134187647 | 0.003288526 | 0.015995306 |
| LGG (n=516) | Neutrophil_CIBERSORT-ABS | 0.169553237 | 0.00019588 | 0.001462622 |
| LGG (n=516) | Neutrophil_MCPCOUNTER | -0.111124861 | 0.015068945 | 0.055404988 |
| LGG (n=516) | Neutrophil_QUANTISEQ | NA | NA | NA |
| LGG (n=516) | Neutrophil_TIMER | 0.594743923 | 4.65E-47 | 3.54E-44 |
| LGG (n=516) | Neutrophil_XCELL | 0.051092232 | 0.264917344 | 0.462061092 |
| LGG (n=516) | NK cell activated_CIBERSORT | 0.019984107 | 0.662969996 | 0.804031179 |
| LGG (n=516) | NK cell activated_CIBERSORT-ABS | 0.198023644 | 1.29E-05 | 0.00013235 |
| LGG (n=516) | NK cell resting_CIBERSORT | 0.1399014 | 0.00217106 | 0.011202865 |
| LGG (n=516) | NK cell resting_CIBERSORT-ABS | 0.168657226 | 0.000211973 | 0.001560172 |
| LGG (n=516) | NK cell_EPIC | 0.299877362 | 2.17E-11 | 5.68E-10 |
| LGG (n=516) | NK cell_MCPCOUNTER | -0.13377854 | 0.003385772 | 0.016433381 |
| LGG (n=516) | NK cell_QUANTISEQ | -0.39726899 | 1.60E-19 | 1.17E-17 |
| LGG (n=516) | NK cell_XCELL | -0.050674503 | 0.268848982 | 0.46671757 |
| LGG (n=516) | Plasmacytoid dendritic cell_XCELL | 0.087337403 | 0.056375173 | 0.153315014 |
| LGG (n=516) | T cell CD4+ (non-regulatory)_QUANTISEQ | 0.16509272 | 0.000289062 | 0.002060186 |
| LGG (n=516) | T cell CD4+ (non-regulatory)_XCELL | -0.037601369 | 0.412088218 | 0.608036033 |
| LGG (n=516) | T cell CD4+ central memory_XCELL | -0.173495665 | 0.000137741 | 0.001075839 |
| LGG (n=516) | T cell CD4+ effector memory_XCELL | 0.0829265 | 0.070076846 | 0.180090805 |
| LGG (n=516) | T cell CD4+ memory activated_CIBERSORT | 0.025042241 | 0.584958733 | 0.747415602 |
| LGG (n=516) | T cell CD4+ memory activated_CIBERSORT-ABS | 0.025630189 | 0.576173368 | 0.739488112 |
| LGG (n=516) | T cell CD4+ memory resting_CIBERSORT | 0.39234525 | 4.86E-19 | 3.42E-17 |
| LGG (n=516) | T cell CD4+ memory resting_CIBERSORT-ABS | 0.49852971 | 2.13E-31 | 3.26E-29 |
| LGG (n=516) | T cell CD4+ memory_XCELL | 0.029591639 | 0.518657825 | 0.695420984 |
| LGG (n=516) | T cell CD4+ naive_CIBERSORT | -0.287526827 | 1.50E-10 | 3.43E-09 |
| LGG (n=516) | T cell CD4+ naive_CIBERSORT-ABS | -0.281216641 | 3.88E-10 | 8.61E-09 |
| LGG (n=516) | T cell CD4+ naive_XCELL | -0.046903449 | 0.306147864 | 0.506821707 |
| LGG (n=516) | T cell CD4+ Th1_XCELL | 0.235323747 | 1.94E-07 | 2.70E-06 |
| LGG (n=516) | T cell CD4+ Th2_XCELL | 0.504671284 | 2.97E-32 | 5.04E-30 |
| LGG (n=516) | T cell CD4+_EPIC | -0.354073599 | 1.45E-15 | 7.00E-14 |
| LGG (n=516) | T cell CD4+_TIMER | 0.503206375 | 4.77E-32 | 7.80E-30 |
| LGG (n=516) | T cell CD8+ central memory_XCELL | -0.11889518 | 0.009271986 | 0.037489293 |
| LGG (n=516) | T cell CD8+ effector memory_XCELL | -0.087969833 | 0.054606396 | 0.149750435 |
| LGG (n=516) | T cell CD8+ naive_XCELL | -0.041841001 | 0.361356508 | 0.561404635 |
| LGG (n=516) | T cell CD8+_CIBERSORT | 0.080023484 | 0.080499806 | 0.199376415 |
| LGG (n=516) | T cell CD8+_CIBERSORT-ABS | 0.162150727 | 0.000371651 | 0.002546477 |
| LGG (n=516) | T cell CD8+_EPIC | -0.324324144 | 3.60E-13 | 1.30E-11 |
| LGG (n=516) | T cell CD8+_MCPCOUNTER | 0.032529774 | 0.477995008 | 0.663137722 |
| LGG (n=516) | T cell CD8+_QUANTISEQ | 0.098299178 | 0.031656997 | 0.098567399 |
| LGG (n=516) | T cell CD8+_TIMER | -0.267529852 | 2.81E-09 | 5.42E-08 |
| LGG (n=516) | T cell CD8+_XCELL | 0.014138716 | 0.757835958 | 0.867587588 |
| LGG (n=516) | T cell follicular helper_CIBERSORT | -0.228397174 | 4.48E-07 | 5.87E-06 |
| LGG (n=516) | T cell follicular helper_CIBERSORT-ABS | -0.070411036 | 0.124222482 | 0.271781215 |
| LGG (n=516) | T cell gamma delta_CIBERSORT | 0.010037801 | 0.826736131 | 0.907896538 |
| LGG (n=516) | T cell gamma delta_CIBERSORT-ABS | 0.010037801 | 0.826736131 | 0.907896538 |
| LGG (n=516) | T cell gamma delta_XCELL | -0.061841769 | 0.177076326 | 0.351491532 |
| LGG (n=516) | T cell NK_XCELL | -0.092234314 | 0.043846356 | 0.127239346 |
| LGG (n=516) | T cell regulatory (Tregs)_CIBERSORT | 0.180262314 | 7.39E-05 | 0.000617382 |
| LGG (n=516) | T cell regulatory (Tregs)_CIBERSORT-ABS | 0.195321819 | 1.70E-05 | 0.000165121 |
| LGG (n=516) | T cell regulatory (Tregs)_QUANTISEQ | 0.036638947 | 0.424165119 | 0.6194967 |
| LGG (n=516) | T cell regulatory (Tregs)_XCELL | -0.190147239 | 2.86E-05 | 0.000262045 |
| GBM (n=153) | B cell memory_CIBERSORT | 0.007793841 | 0.927977235 | 0.963118323 |
| GBM (n=153) | B cell memory_CIBERSORT-ABS | -0.006805257 | 0.93709262 | 0.968188019 |
| GBM (n=153) | B cell memory_XCELL | 0.074200332 | 0.388839608 | 0.58842888 |
| GBM (n=153) | B cell naive_CIBERSORT | -0.124117121 | 0.148447651 | 0.310674394 |
| GBM (n=153) | B cell naive_CIBERSORT-ABS | -0.15318199 | 0.073923982 | 0.187140523 |
| GBM (n=153) | B cell naive_XCELL | -0.19113192 | 0.025265991 | 0.083217169 |
| GBM (n=153) | B cell plasma_CIBERSORT | -0.174840617 | 0.041005527 | 0.121163524 |
| GBM (n=153) | B cell plasma_CIBERSORT-ABS | -0.218146389 | 0.010441276 | 0.041520175 |
| GBM (n=153) | B cell plasma_XCELL | 0.025347462 | 0.768747757 | 0.876306679 |
| GBM (n=153) | B cell_EPIC | -0.322597294 | 0.000120742 | 0.000962779 |
| GBM (n=153) | B cell_MCPCOUNTER | -0.280893064 | 0.000884675 | 0.005384518 |
| GBM (n=153) | B cell_QUANTISEQ | -0.124787755 | 0.146249916 | 0.307057737 |
| GBM (n=153) | B cell_TIMER | -0.148814113 | 0.082644807 | 0.203477829 |
| GBM (n=153) | B cell_XCELL | -0.116257576 | 0.176092636 | 0.350424346 |
| GBM (n=153) | Cancer associated fibroblast_EPIC | 0.079027673 | 0.35864163 | 0.55906422 |
| GBM (n=153) | Cancer associated fibroblast_MCPCOUNTER | 0.231170779 | 0.006569587 | 0.028393768 |
| GBM (n=153) | Cancer associated fibroblast_TIDE | 0.147853126 | 0.084670035 | 0.207275501 |
| GBM (n=153) | Cancer associated fibroblast_XCELL | 0.220358486 | 0.009667955 | 0.038952665 |
| GBM (n=153) | Class-switched memory B cell_XCELL | -0.297763979 | 0.000409613 | 0.002773374 |
| GBM (n=153) | Common lymphoid progenitor_XCELL | 0.485083938 | 1.89E-09 | 3.74E-08 |
| GBM (n=153) | Common myeloid progenitor_XCELL | 0.126994109 | 0.139193072 | 0.297010113 |
| GBM (n=153) | Endothelial cell_EPIC | -0.176782088 | 0.038778693 | 0.115704092 |
| GBM (n=153) | Endothelial cell_MCPCOUNTER | -0.148376664 | 0.083561839 | 0.205183765 |
| GBM (n=153) | Endothelial cell_XCELL | -0.00338668 | 0.968669566 | 0.983932668 |
| GBM (n=153) | Eosinophil_CIBERSORT | 0.003585372 | 0.966832461 | 0.983002866 |
| GBM (n=153) | Eosinophil_CIBERSORT-ABS | 0.001467079 | 0.986425135 | 0.992497524 |
| GBM (n=153) | Eosinophil_XCELL | -0.413446707 | 5.13E-07 | 6.65E-06 |
| GBM (n=153) | Granulocyte-monocyte progenitor_XCELL | -0.045412554 | 0.598233866 | 0.756594751 |
| GBM (n=153) | Hematopoietic stem cell_XCELL | 0.01320477 | 0.878281255 | 0.935076368 |
| GBM (n=153) | Macrophage M0_CIBERSORT | 0.12687889 | 0.139555054 | 0.297366611 |
| GBM (n=153) | Macrophage M0_CIBERSORT-ABS | 0.111806282 | 0.193343812 | 0.373390137 |
| GBM (n=153) | Macrophage M1_CIBERSORT | 0.127035068 | 0.139064563 | 0.296874302 |
| GBM (n=153) | Macrophage M1_CIBERSORT-ABS | 0.039956108 | 0.64295275 | 0.789799983 |
| GBM (n=153) | Macrophage M1_QUANTISEQ | 0.112894388 | 0.189017603 | 0.367048608 |
| GBM (n=153) | Macrophage M1_XCELL | 0.188908237 | 0.027049691 | 0.087433923 |
| GBM (n=153) | Macrophage M2_CIBERSORT | 0.092701399 | 0.28129171 | 0.480220871 |
| GBM (n=153) | Macrophage M2_CIBERSORT-ABS | -0.120731303 | 0.159925604 | 0.326644 |
| GBM (n=153) | Macrophage M2_QUANTISEQ | 0.046901447 | 0.586278378 | 0.748089249 |
| GBM (n=153) | Macrophage M2_TIDE | 0.128501937 | 0.134521592 | 0.289063533 |
| GBM (n=153) | Macrophage M2_XCELL | -0.053499872 | 0.534660664 | 0.706042083 |
| GBM (n=153) | Macrophage_EPIC | 0.095953803 | 0.264680891 | 0.462061092 |
| GBM (n=153) | Macrophage_TIMER | -0.009882149 | 0.908753805 | 0.952453897 |
| GBM (n=153) | Macrophage_XCELL | 0.153038591 | 0.074197981 | 0.187555662 |
| GBM (n=153) | Macrophage/Monocyte_MCPCOUNTER | -0.348353 | 3.03E-05 | 0.000274881 |
| GBM (n=153) | Mast cell activated_CIBERSORT | 0.010323365 | 0.90469867 | 0.950645285 |
| GBM (n=153) | Mast cell activated_CIBERSORT-ABS | 0.011316696 | 0.895578287 | 0.945835191 |
| GBM (n=153) | Mast cell resting_CIBERSORT | 0.030324649 | 0.725011018 | 0.846315579 |
| GBM (n=153) | Mast cell resting_CIBERSORT-ABS | -0.002316356 | 0.978568316 | 0.988571931 |
| GBM (n=153) | Mast cell_XCELL | -0.090723561 | 0.291728029 | 0.492344833 |
| GBM (n=153) | MDSC_TIDE | 0.379069126 | 4.93E-06 | 5.46E-05 |
| GBM (n=153) | Monocyte_CIBERSORT | -0.207534082 | 0.014956842 | 0.055207633 |
| GBM (n=153) | Monocyte_CIBERSORT-ABS | -0.243167408 | 0.004193469 | 0.019525441 |
| GBM (n=153) | Monocyte_MCPCOUNTER | -0.348353 | 3.03E-05 | 0.000274881 |
| GBM (n=153) | Monocyte_QUANTISEQ | -0.183704761 | 0.031647888 | 0.098567399 |
| GBM (n=153) | Monocyte_XCELL | 0.184899458 | 0.030537356 | 0.095536211 |
| GBM (n=153) | Myeloid dendritic cell activated_CIBERSORT | 0.060594065 | 0.481816728 | 0.666603818 |
| GBM (n=153) | Myeloid dendritic cell activated_CIBERSORT-ABS | 0.062028496 | 0.471484097 | 0.659530169 |
| GBM (n=153) | Myeloid dendritic cell activated_XCELL | -0.116438848 | 0.17541498 | 0.349683957 |
| GBM (n=153) | Myeloid dendritic cell resting_CIBERSORT | -0.019799671 | 0.818365084 | 0.905824248 |
| GBM (n=153) | Myeloid dendritic cell resting_CIBERSORT-ABS | -0.013262868 | 0.877749877 | 0.935076368 |
| GBM (n=153) | Myeloid dendritic cell_MCPCOUNTER | 0.186663689 | 0.028958034 | 0.092042306 |
| GBM (n=153) | Myeloid dendritic cell_QUANTISEQ | -0.138063102 | 0.107639257 | 0.246312935 |
| GBM (n=153) | Myeloid dendritic cell_TIMER | -0.126400368 | 0.141066095 | 0.299332182 |
| GBM (n=153) | Myeloid dendritic cell_XCELL | 0.137577452 | 0.10889465 | 0.248089007 |
| GBM (n=153) | Neutrophil_CIBERSORT | -0.015908596 | 0.853612689 | 0.922752049 |
| GBM (n=153) | Neutrophil_CIBERSORT-ABS | -0.053789335 | 0.53244928 | 0.704952373 |
| GBM (n=153) | Neutrophil_MCPCOUNTER | -0.484283026 | 2.02E-09 | 3.99E-08 |
| GBM (n=153) | Neutrophil_QUANTISEQ | 0.045580987 | 0.596875913 | 0.755712601 |
| GBM (n=153) | Neutrophil_TIMER | -0.029855152 | 0.729101501 | 0.84870071 |
| GBM (n=153) | Neutrophil_XCELL | -0.019514242 | 0.820939263 | 0.906936763 |
| GBM (n=153) | NK cell activated_CIBERSORT | -0.061422416 | 0.475835068 | 0.662176073 |
| GBM (n=153) | NK cell activated_CIBERSORT-ABS | -0.096695797 | 0.260986996 | 0.457524775 |
| GBM (n=153) | NK cell resting_CIBERSORT | -0.038542608 | 0.654757842 | 0.79930838 |
| GBM (n=153) | NK cell resting_CIBERSORT-ABS | -0.074199769 | 0.388843217 | 0.58842888 |
| GBM (n=153) | NK cell_EPIC | 0.123134949 | 0.151711209 | 0.31546268 |
| GBM (n=153) | NK cell_MCPCOUNTER | -0.320583176 | 0.000133855 | 0.001047271 |
| GBM (n=153) | NK cell_QUANTISEQ | -0.393106343 | 2.02E-06 | 2.38E-05 |
| GBM (n=153) | NK cell_XCELL | 0.142754681 | 0.096087396 | 0.22599795 |
| GBM (n=153) | Plasmacytoid dendritic cell_XCELL | 0.099847517 | 0.245690477 | 0.43772881 |
| GBM (n=153) | T cell CD4+ (non-regulatory)_QUANTISEQ | 0.149199101 | 0.081844439 | 0.201615713 |
| GBM (n=153) | T cell CD4+ (non-regulatory)_XCELL | 0.049907887 | 0.562477487 | 0.728277075 |
| GBM (n=153) | T cell CD4+ central memory_XCELL | -0.249522287 | 0.003277024 | 0.015973313 |
| GBM (n=153) | T cell CD4+ effector memory_XCELL | -0.080024869 | 0.352590317 | 0.554390798 |
| GBM (n=153) | T cell CD4+ memory activated_CIBERSORT | 0.133204734 | 0.120719809 | 0.266281719 |
| GBM (n=153) | T cell CD4+ memory activated_CIBERSORT-ABS | 0.13429849 | 0.117672678 | 0.261465243 |
| GBM (n=153) | T cell CD4+ memory resting_CIBERSORT | 0.011179211 | 0.896839835 | 0.946905635 |
| GBM (n=153) | T cell CD4+ memory resting_CIBERSORT-ABS | -0.036764793 | 0.669727339 | 0.808280438 |
| GBM (n=153) | T cell CD4+ memory_XCELL | 0.254475238 | 0.002692467 | 0.013557121 |
| GBM (n=153) | T cell CD4+ naive_CIBERSORT | -0.168859003 | 0.048549957 | 0.136999478 |
| GBM (n=153) | T cell CD4+ naive_CIBERSORT-ABS | -0.169613711 | 0.047538746 | 0.134644085 |
| GBM (n=153) | T cell CD4+ naive_XCELL | -0.157256592 | 0.066474274 | 0.173858716 |
| GBM (n=153) | T cell CD4+ Th1_XCELL | -0.177188837 | 0.038325265 | 0.114650156 |
| GBM (n=153) | T cell CD4+ Th2_XCELL | 0.561903748 | 9.06E-13 | 2.94E-11 |
| GBM (n=153) | T cell CD4+_EPIC | -0.504809147 | 3.17E-10 | 7.08E-09 |
| GBM (n=153) | T cell CD4+_TIMER | -0.354922494 | 2.08E-05 | 0.000197988 |
| GBM (n=153) | T cell CD8+ central memory_XCELL | 0.323109258 | 0.000117605 | 0.000946008 |
| GBM (n=153) | T cell CD8+ effector memory_XCELL | -0.097960722 | 0.25477137 | 0.449706348 |
| GBM (n=153) | T cell CD8+ naive_XCELL | 0.230489102 | 0.006734991 | 0.028917501 |
| GBM (n=153) | T cell CD8+_CIBERSORT | 0.195476166 | 0.022070292 | 0.074881933 |
| GBM (n=153) | T cell CD8+_CIBERSORT-ABS | 0.102224139 | 0.234574885 | 0.424493108 |
| GBM (n=153) | T cell CD8+_EPIC | -0.123386511 | 0.150870219 | 0.314307234 |
| GBM (n=153) | T cell CD8+_MCPCOUNTER | 0.036483861 | 0.672104934 | 0.809092551 |
| GBM (n=153) | T cell CD8+_QUANTISEQ | 0.291181606 | 0.000556281 | 0.003591113 |
| GBM (n=153) | T cell CD8+_TIMER | 0.290320004 | 0.000578708 | 0.003720152 |
| GBM (n=153) | T cell CD8+_XCELL | 0.079086247 | 0.358284405 | 0.55906422 |
| GBM (n=153) | T cell follicular helper_CIBERSORT | 0.000449072 | 0.995844566 | 0.997588221 |
| GBM (n=153) | T cell follicular helper_CIBERSORT-ABS | -0.100948082 | 0.240498484 | 0.4316712 |
| GBM (n=153) | T cell gamma delta_CIBERSORT | 0.072034598 | 0.402870653 | 0.601115404 |
| GBM (n=153) | T cell gamma delta_CIBERSORT-ABS | 0.072868867 | 0.397430547 | 0.596602038 |
| GBM (n=153) | T cell gamma delta_XCELL | 0.022794836 | 0.791474467 | 0.890287204 |
| GBM (n=153) | T cell NK_XCELL | -0.331112624 | 7.75E-05 | 0.000645746 |
| GBM (n=153) | T cell regulatory (Tregs)_CIBERSORT | 0.073556246 | 0.392981362 | 0.592644381 |
| GBM (n=153) | T cell regulatory (Tregs)_CIBERSORT-ABS | 0.061959055 | 0.471981498 | 0.659822638 |
| GBM (n=153) | T cell regulatory (Tregs)_QUANTISEQ | 0.059562406 | 0.489322712 | 0.671016281 |
| GBM (n=153) | T cell regulatory (Tregs)_XCELL | -0.233906326 | 0.005941578 | 0.026148655 |

**Supplementary Table S8:** Correlation analysis between *GPX7* and related markers of immune cells.

|  |  | **Low-Grade Glioma** | | | | **Glioblastoma** | | | |
| --- | --- | --- | --- | --- | --- | --- | --- | --- | --- |
| **Description** | **Gene (Also known as)** | **Without Adjustment** | | **Purity Adjustment** | | **Without Adjustment** | | **Purity Adjustment** | |
|  |  | Correlation | Adjusted P-value | Correlation | Adjusted P-value | Correlation | Adjusted P-value | Correlation | Adjusted P-value |
| **CD8^+^T cell** | CD8A | 0.1333 | **7.08E-03** | 0.1304 | **1.40E-02** | -0.0389 | 7.25E-01 | 0.0129 | 9.24E-01 |
|  | CD8B | 0.0314 | 5.83E-01 | 0.0325 | 6.15E-01 | -0.0378 | 7.34E-01 | 0.0365 | 7.79E-01 |
| **T cell (general)** | CD3E | 0.3661 | **2.10E-16** | 0.3691 | **3.49E-15** | -0.0491 | 6.52E-01 | 0.0406 | 7.52E-01 |
|  | CD3D | 0.3460 | **1.19E-14** | 0.3499 | **1.24E-13** | 0.0011 | 9.91E-01 | 0.1407 | 1.96E-01 |
|  | CD2 | 0.3853 | **3.34E-18** | 0.3871 | **9.20E-17** | -0.0153 | 8.95E-01 | 0.0954 | 4.12E-01 |
| **B cell** | CD19 | 0.3242 | **7.12E-13** | 0.3117 | **7.37E-11** | -0.1047 | 2.97E-01 | -0.0686 | 5.68E-01 |
|  | CD79A | 0.3192 | **1.77E-12** | 0.3421 | **4.80E-13** | 0.0263 | 8.17E-01 | 0.0694 | 5.64E-01 |
| **Monocyte** | CD86 | 0.5223 | **4.81E-35** | 0.5429 | **4.20E-35** | -0.2143 | **2.01E-02** | -0.0728 | 5.44E-01 |
|  | CD115 (CSF1R) | 0.4315 | **5.68E-23** | 0.4548 | **1.10E-23** | -0.3416 | **7.31E-05** | -0.2543 | **9.43E-03** |
| **TAM** | CCL2 | 0.3021 | **3.16E-11** | 0.2817 | **5.90E-09** | -0.0198 | 8.66E-01 | 0.1564 | 1.44E-01 |
|  | CD68 | 0.5018 | **6.25E-32** | 0.5020 | **1.66E-29** | -0.2582 | **3.96E-03** | -0.1166 | 2.98E-01 |
|  | IL10 | 0.3910 | **9.40E-19** | 0.3780 | **5.82E-16** | -0.0820 | 4.25E-01 | 0.1312 | 2.32E-01 |
| **M1 Macrophage** | IRF5 | 0.4949 | **4.99E-31** | 0.5264 | **6.68E-33** | -0.3640 | **1.91E-05** | -0.3258 | **5.49E-04** |
|  | iNOS(NOS2) | -0.0386 | 4.92E-01 | -0.0231 | 7.29E-01 | 0.1076 | 2.80E-01 | 0.0724 | 5.47E-01 |
|  | COX2(PTGS2) | 0.0557 | 3.07E-01 | 0.0459 | 4.66E-01 | -0.0220 | 8.51E-01 | 0.0788 | 5.10E-01 |
| **M2 Macrophage** | CD163 | 0.3646 | **2.88E-16** | 0.3537 | **6.33E-14** | -0.0332 | 7.65E-01 | 0.1419 | 1.92E-01 |
|  | VSIG4 | 0.4756 | **2.04E-28** | 0.4754 | **3.76E-26** | -0.0741 | 4.71E-01 | 0.1183 | 2.90E-01 |
|  | MS4A4A | 0.4251 | **2.83E-22** | 0.4249 | **2.29E-20** | -0.0039 | 9.72E-01 | 0.2127 | **3.55E-02** |
| **Neutrophils** | CD66b(CEACAM8) | 0.0607 | 2.62E-01 | 0.0433 | 4.96E-01 | -0.0975 | 3.35E-01 | -0.0698 | 5.62E-01 |
|  | CD11b(ITGAM) | 0.4749 | **2.42E-28** | 0.5016 | **1.70E-29** | -0.3683 | **1.46E-05** | -0.2723 | **5.00E-03** |
|  | CCR7 | 0.1843 | **1.14E-04** | 0.1841 | **2.98E-04** | -0.0528 | 6.25E-01 | 0.0450 | 7.20E-01 |
| **NK cell** | KIR2DL1 | 0.0353 | 5.34E-01 | 0.0387 | 5.44E-01 | -0.1252 | 2.05E-01 | -0.1108 | 3.28E-01 |
|  | KIR2DL3 | 0.1687 | **4.63E-04** | 0.1767 | **5.51E-04** | -0.2283 | **1.25E-02** | -0.2060 | **4.31E-02** |
|  | KIR2DL4 | 0.3530 | **3.03E-15** | 0.3482 | **1.67E-13** | -0.2023 | **2.94E-02** | -0.1698 | 1.08E-01 |
|  | KIR3DL1 | -0.0071 | 9.10E-01 | -0.0056 | 9.38E-01 | -0.0432 | 6.98E-01 | -0.0552 | 6.51E-01 |
|  | KIR3DL2 | 0.1711 | **3.74E-04** | 0.1750 | **6.28E-04** | -0.1230 | 2.13E-01 | -0.1155 | 3.02E-01 |
|  | KIR3DL3 | 0.0503 | 3.61E-01 | 0.0706 | 2.28E-01 | -0.1171 | 2.39E-01 | -0.1415 | 1.93E-01 |
|  | KIR2DS4 | 0.1165 | **2.06E-02** | 0.1190 | **2.68E-02** | -0.2013 | **3.01E-02** | -0.1522 | 1.57E-01 |
| **Dendritic cell** | HLA-DPB1 | 0.4948 | **4.99E-31** | 0.5003 | **2.41E-29** | -0.1456 | 1.34E-01 | -0.0232 | 8.62E-01 |
|  | HLA-DQB1 | 0.4119 | **6.74E-21** | 0.4103 | **6.75E-19** | -0.0624 | 5.52E-01 | 0.0400 | 7.55E-01 |
|  | HLA-DRA | 0.5342 | **9.85E-37** | 0.5375 | **1.83E-34** | -0.0874 | 3.92E-01 | 0.0798 | 5.05E-01 |
|  | HLA-DPA1 | 0.4944 | **5.42E-31** | 0.4982 | **4.31E-29** | -0.1302 | 1.85E-01 | -0.0443 | 7.24E-01 |
|  | BDCA-1(CD1C) | 0.2495 | **6.63E-08** | 0.2430 | **7.60E-07** | 0.0589 | 5.77E-01 | 0.1558 | 1.45E-01 |
|  | BDCA-4(NRP1) | 0.2089 | **9.12E-06** | 0.1930 | **1.37E-04** | -0.0077 | 9.49E-01 | 0.0579 | 6.36E-01 |
| **CD4^+^T cell** | CD4 | 0.5056 | **1.80E-32** | 0.5202 | **4.41E-32** | -0.3058 | **4.74E-04** | -0.1970 | 5.44E-02 |
|  | CD45RA (PTPRC) | 0.5248 | **2.45E-35** | 0.5401 | **8.79E-35** | -0.3058 | **4.74E-04** | -0.1572 | 1.42E-01 |
|  | CD11c(ITGAX) | 0.4146 | **3.69E-21** | 0.4204 | **6.35E-20** | -0.4378 | **1.06E-07** | -0.4051 | **7.73E-06** |
| **Th1** | T-bet(TBX21) | 0.3781 | **1.69E-17** | 0.3685 | **3.76E-15** | -0.0228 | 8.44E-01 | -0.0841 | 4.79E-01 |
|  | STAT4 | -0.1924 | **5.20E-05** | -0.1810 | **3.86E-04** | -0.2009 | **3.04E-02** | -0.1455 | 1.79E-01 |
|  | STAT1 | 0.4154 | **3.16E-21** | 0.3988 | **8.32E-18** | -0.1417 | 1.46E-01 | -0.1800 | 8.41E-02 |
|  | IFN-γ(IFNG) | 0.1969 | **3.29E-05** | 0.2040 | **4.82E-05** | -0.0681 | 5.15E-01 | -0.0323 | 8.04E-01 |
|  | TNF-α(TNF) | 0.1463 | **2.80E-03** | 0.1228 | **2.17E-02** | -0.1770 | 6.19E-02 | -0.1194 | 2.85E-01 |
| **Th2** | GATA3 | 0.4138 | **4.29E-21** | 0.4061 | **1.70E-18** | 0.0016 | 9.89E-01 | 0.0101 | 9.40E-01 |
|  | STAT6 | 0.2092 | **8.90E-06** | 0.2527 | **2.42E-07** | -0.3254 | **1.75E-04** | -0.2575 | **8.50E-03** |
|  | STAT5A | 0.5728 | **2.92E-43** | 0.5871 | **2.99E-42** | -0.2229 | **1.51E-02** | -0.1665 | 1.16E-01 |
|  | IL13 | 0.0472 | 3.94E-01 | 0.0506 | 4.14E-01 | 0.0630 | 5.49E-01 | -0.0179 | 8.94E-01 |
| **Tfh** | BCL6 | 0.2152 | **4.53E-06** | 0.0506 | 4.14E-01 | -0.3001 | **6.25E-04** | -0.3369 | **3.24E-04** |
|  | IL21 | 0.1096 | **3.03E-02** | 0.1004 | 6.99E-02 | -0.1769 | 6.19E-02 | -0.1236 | 2.66E-01 |
| **Th17** | STAT3 | 0.5011 | **7.13E-32** | 0.4655 | **6.20E-25** | -0.1768 | 6.20E-02 | -0.2018 | **4.84E-02** |
|  | IL-17A | 0.0171 | 7.78E-01 | 0.0031 | 9.66E-01 | -0.1186 | 2.33E-01 | -0.0929 | 4.26E-01 |
| **Treg** | FOXP3 | -0.1711 | **3.73E-04** | -0.1535 | **3.16E-03** | -0.2962 | **7.55E-04** | -0.2787 | **3.93E-03** |
|  | CCR8 | 0.1747 | **2.70E-04** | 0.1834 | **3.14E-04** | 0.0562 | 5.97E-01 | 0.1195 | 2.85E-01 |
|  | STAT5B | 0.1447 | **3.18E-03** | 0.1392 | **8.24E-03** | -0.1640 | 8.56E-02 | -0.2798 | **3.75E-03** |
|  | TGFβ(TGFB1) | 0.5302 | **3.79E-36** | 0.5297 | **2.50E-33** | -0.1301 | 1.85E-01 | -0.2798 | **3.75E-03** |
| **T cell exhaustion** | PD-1(PDCD1) | 0.3540 | **2.58E-15** | 0.3280 | **5.20E-12** | -0.1291 | 1.89E-01 | -0.0866 | 4.64E-01 |
|  | CTLA4 | 0.2811 | **8.40E-10** | 0.2779 | **9.70E-09** | -0.2136 | **2.06E-02** | -0.1277 | 2.49E-01 |
|  | TIGIT | -0.0977 | 5.76E-02 | -0.1203 | **2.50E-02** | -0.1592 | 9.65E-02 | -0.1223 | 2.71E-01 |
|  | TIM-3(HAVCR2) | 0.5583 | **1.02E-40** | -0.1203 | **2.50E-02** | -0.2504 | **5.46E-03** | -0.1245 | 2.62E-01 |
|  | LAG3 | 0.2785 | **1.24E-09** | 0.2728 | **1.92E-08** | -0.0781 | 4.47E-01 | -0.1157 | 3.02E-01 |

**Supplementary Table S9:** Correlations of *GPX7* with chemokines and their respective receptors via GEPIA.

|  |  | **LGG** | | **GBM** | |
| --- | --- | --- | --- | --- | --- |
|  |  | **Spearman's correlation coefficient (ρ)** | **P-value** | **Spearman's correlation coefficient (ρ)** | **P-value** |
| **Chemokines** | CCL1 | −0.34 | 1.7e−15 | −0.026 | 0.74 |
|  | CCL2 | 0.30 | 3.5e−12 | 0.014 | 0.86 |
|  | CCL3 | 0.019 | 0.66 | −0.17 | 0.03 |
|  | CCL4 | 0.054 | 0.22 | −0.23 | 0.0037 |
|  | CCL5 | 0.38 | 4.7e−19 | −0.083 | 0.29 |
|  | CCL7 | 0.038 | 0.39 | −0.037 | 0.64 |
|  | CCL8 | 0.18 | 4.5e−05 | 0.01 | 0.9 |
|  | CCL11 | −0.018 | 0.68 | 0.12 | 0.12 |
|  | CCL13 | −0.067 | 0.13 | −0.078 | 0.32 |
|  | CCL14 | 0.034 | 0.45 | 0.025 | 0.75 |
|  | CCL15 | 0.015 | 0.73 | 0.021 | 0.79 |
|  | CCL16 | −0.0046 | 0.92 | 0.11 | 0.15 |
|  | CCL17 | 0.24 | 4.7e−08 | −0.0085 | 0.91 |
|  | CCL18 | −0.098 | 0.026 | −0.014 | 0.86 |
|  | CCL19 | 0.039 | 0.37 | −0.068 | 0.39 |
|  | CCL20 | 0.31 | 5.3e−13 | 0.014 | 0.86 |
|  | CCL21 | 0.071 | 0.11 | −0.056 | 0.48 |
|  | CCL22 | 0.33 | 2e−14 | −0.063 | 0.42 |
|  | CCL23 | 0.24 | 4.7e−08 | −0.067 | 0.4 |
|  | CCL24 | −0.055 | 0.21 | 0.13 | 0.1 |
|  | CCL25 | 0.33 | 3.1e−14 | 0.00098 | 0.99 |
|  | CCL26 | 0.12 | 0.006 | 0.22 | 0.0048 |
|  | CCL27 | 0.13 | 0.0039 | −0.033 | 0.68 |
|  | CCL28 | −0.16 | 0.00027 | −0.12 | 0.13 |
|  | CX3CL1 | −0.16 | 0.00034 | −0.22 | 0.0044 |
|  | CXCL1 | 4.2e−05 | 1 | 0.02 | 0.8 |
|  | CXCL2 | −0.0092 | 0.84 | −0.1 | 0.2 |
|  | CXCL3 | −0.018 | 0.67 | 0.015 | 0.85 |
|  | CXCL5 | −0.22 | 3.7e−07 | −0.085 | 0.28 |
|  | CXCL6 | 0.025 | 0.57 | 0.055 | 0.49 |
|  | CXCL8 | 0.07 | 0.11 | 0.027 | 0.73 |
|  | CXCL9 | 0.4 | 8.2e−21 | 0.055 | 0.49 |
|  | CXCL10 | 0.42 | 5.8e−24 | 0.15 | 0.052 |
|  | CXCL11 | 0.38 | 6.2e−19 | 0.19 | 0.013 |
|  | CXCL12 | 0.13 | 0.0021 | −0.16 | 0.039 |
|  | CXCL13 | −0.17 | 0.00011 | 0.14 | 0.082 |
|  | CXCL14 | −0.14 | 0.0011 | −0.14 | 0.085 |
|  | CXCL16 | 0.45 | 9.8e−28 | −0.17 | 0.033 |
|  | CXCL17 | 0.043 | 0.33 | −0.014 | 0.86 |
|  | XCL1 | 0.26 | 9.7e−10 | 0.059 | 0.46 |
|  | XCL2 | 0.27 | 3.4e−10 | 0.029 | 0.72 |
| **Chemokine receptors** | CCR1 | 0.5 | 2.6e−34 | −0.23 | 0.0026 |
|  | CCR2 | 0.38 | 3.5e−19 | −0.016 | 0.84 |
|  | CCR3 | 0.28 | 1.4e−10 | −0.16 | 0.046 |
|  | CCR4 | 0.29 | 8.7e−12 | 0.0092 | 0.91 |
|  | CCR5 | 0.55 | 9.4e−43 | −0.21 | 0.0063 |
|  | CCR6 | −0.017 | 0.7 | −0.11 | 0.16 |
|  | CCR7 | 0.21 | 1.1e−06 | −0.049 | 0.53 |
|  | CCR8 | 0.2 | 5.9e−06 | 0.021 | 0.79 |
|  | CCR9 | −0.029 | 0.5 | −0.15 | 0.065 |
|  | CCR10 | 0.13 | 0.0024 | −0.1 | 0.2 |
|  | CXCR1 | 0.11 | 0.011 | −0.17 | 0.032 |
|  | CXCR2 | 0.46 | 4e−29 | −0.24 | 0.0022 |
|  | CXCR3 | 0.42 | 9.9e−24 | −0.095 | 0.23 |
|  | CXCR4 | 0.34 | 6.9e−16 | 0.055 | 0.49 |
|  | CXCR5 | 0.082 | 0.061 | −0.14 | 0.072 |
|  | CXCR6 | 0.39 | 5.1e−20 | −0.043 | 0.59 |
|  | XCR1 | 0.29 | 2.3e−11 | −0.087 | 0.27 |
|  | CX3CR1 | 0.39 | 7.4e−20 | −0.27 | 0.00052 |
